# Harnessing asymmetric N-heterocyclic carbene ligands to optimise SABRE hyperpolarisation†
†Electronic supplementary information (ESI) available: NMR raw data can be found at https://doi.org/10.15124/42187464-6f06-4805-a487-9b469b7a15e4. CCDC 1848485. For ESI and crystallographic data in CIF or other electronic format see DOI: 10.1039/c8cy01214h


**DOI:** 10.1039/c8cy01214h

**Published:** 2018-09-03

**Authors:** Chin Min Wong, Marianna Fekete, Rhianna Nelson-Forde, Mark R. D. Gatus, Peter J. Rayner, Adrian C. Whitwood, Simon B. Duckett, Barbara A. Messerle

**Affiliations:** a School of Chemistry , University of New South Wales , Sydney 2052 , Australia; b Department of Molecular Sciences , Macquarie University , North Ryde 2109 , Australia . Email: barbara.messerle@mq.edu.au; c Centre for Hyperpolarisation in Magnetic Resonance , York Science Park , University of York , Heslington , York YO10 5NY , UK . Email: simon.duckett@york.ac.uk

## Abstract

The catalytic signal amplification by reversible exchange process has become widely used for the hyperpolarisation of small molecules to improve their magnetic resonance detectability. It harnesses the latent polarisation of *para*hydrogen, and involves the formation of a labile metal complex that often contains an N-heterocyclic carbene (NHC) ligand (*e.g.* [Ir(H)_2_(NHC)(pyridine)_3_]Cl), which act as a polarisation transfer catalyst. Unfortunately, if the target molecule is too bulky, binding to the catalyst is poor and the hyperpolarisation yield is therefore low. We illustrate here the behaviour of a series of asymmetric NHC containing catalysts towards 3,4- and 3,5-lutidine in order to show how catalyst design can be used to dramatically improve the outcome of this catalytic process for sterically encumbered ligands.

## Abstract 2

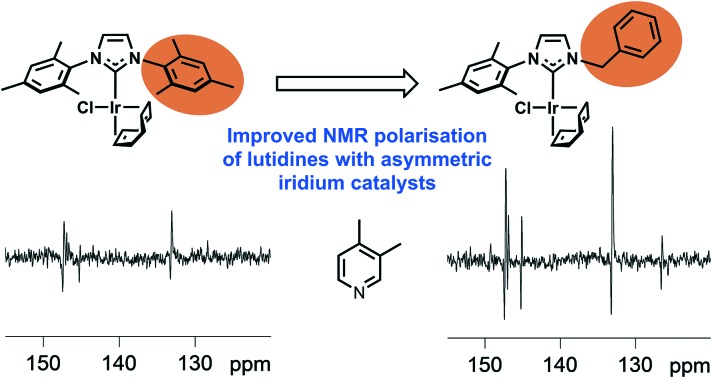
The catalytic signal amplification by reversible exchange process is used widely to improve the magnetic resonance detectability of small molecules by hyperpolarisation.

## Introduction

N-Heterocyclic carbenes (NHCs) are used widely as ligands in an array of chemical transformations such as hydrogenation, hydrogen transfer, hydroformylation, hydrosilylation, oxidation, isomerisation, telomerisation and various C–C bond forming reactions.^1^–^4^ By varying the N-bound substituents of classic imidazole based NHCs it is possible to diversify their electronic and steric properties to control reactivity.^5^ For the free NHCs themselves, bulky substituents provide kinetic stabilisation whilst opening the carbene bond angle favours the triplet rather than the electronic singlet state formation.^6^,^7^ When an NHC is bound to a metal complex the steric bulk of the NHC can be assessed *via* its percent buried volume^8^–^10^ while the electronic effect is based on Tolman's electronic parameter.^11^ Both affect catalytic reactivity.

**Scheme 1:**
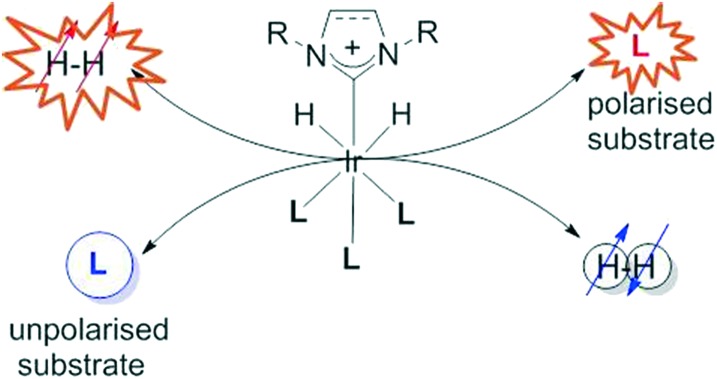
Conceptual representation of the signal amplification by reversible exchange process which achieves the catalytic hyperpolarisation of a substrate (l) *via* polarisation transfer within the iridium catalyst from a pair of protons that were previously located in a molecule of *p*-H_2_.

Here we target a novel form of catalysis, that is abbreviated to SABRE, signal amplification by reversible exchange.^12^ This process takes the latent magnetism of *para*hydrogen (*p*-H_2_) and transfers it into a new material according to the process that is outlined in Scheme 1. Unlike a normal catalytic reaction, which chemically functionalises a substrate, SABRE involves the catalytic transfer of polarisation into a hyperpolarisation target (l) from what is effectively *p*-H_2_. The reversible addition of *p*-H_2_ is therefore important, as it places the source of hyperpolarisation within the coordination sphere of the metal complex. Hence, the ligand exchange rates of the catalyst are important factors when considering how the catalyst works.^12^–^14^

When this process happens in low magnetic field, the effects of chemical shift and coupling become important. Once a second order spin system is created, it becomes possible to transfer the *p*-H_2_ derived hydride ligand polarisation into the NMR active nuclei and then to a reversibly bound ligand through the complex's scalar coupling network.^15^ The importance of this approach stems from the fact that the related hyperpolarisation method, dynamic nuclear polarisation (DNP),^16^ takes materials such as pyruvate and enhances their Magnetic Resonance Imaging (MRI) detectability to the point where they can be seen *in vivo*. In the case of pyruvate,^17^ the subsequent assessment of its metabolism has proven to be diagnostic of human cancer.^17^–^20^ Another approach using hyperpolarised ^129^Xe has progressed to the point it is used for the diagnosis of diseases of the lung in humans.^21^ Hence, if SABRE catalysts can be optimised sufficiently, there is an expectation that a simple low cost route to hyperpolarised agents for disease diagnosis in humans would be possible. The benefits of SABRE for analytical science are however also likely to be substantial.^22^–^25^

**Scheme 2:**
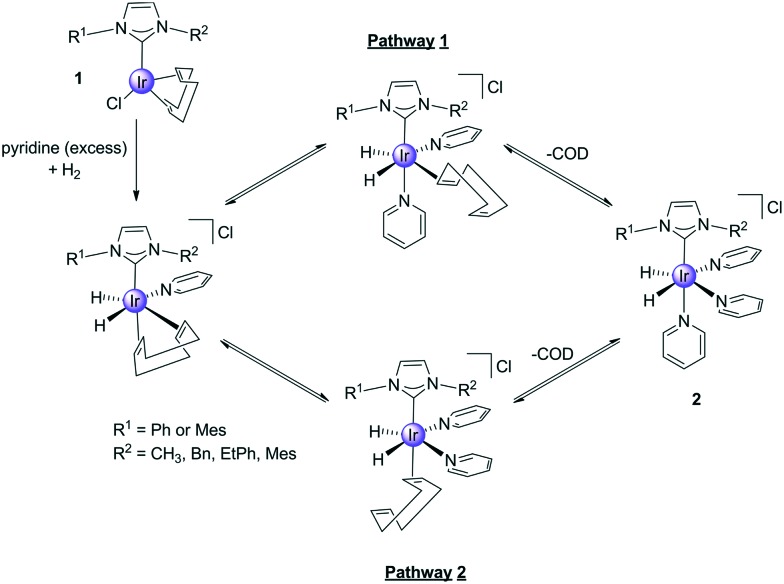
Route to the formation of the active SABRE catalyst **2***via* the reaction of **1** with H_2_ and pyridine. The COD is converted first the COE and COA during this process.

It is common to use precatalysts based on the [Ir(NHC)(COD)Cl] (**1**) motif in SABRE, and they react according to Scheme 2 to form active polarisation transfer catalysts such as [Ir(H)_2_(NHC)(py)_3_]Cl (**2**). During this process cyclooctadiene (COD) is first converted into cyclooctene (COE) and then cyclooctane (COA) and hence whilst the reaction steps shown are reversible, in practice **2** is stable.^26^ The ligand exchange rate of the substrate, in this case pyridine (py), also influences the efficiency of catalytic polarisation transfer because upon ligand loss the propagating spin–spin coupling framework within the catalyst is lost. Ideally, the lifetime of this complex needs to be commensurate with these small scalar couplings that are responsible for hyperpolarisation transfer.^26^,^27^

It was found that iridium catalysts containing NHCs^28^,^29^ were more active than their mono phosphine counterparts because their lifetimes were closer to the optimum.^30^ Whilst other iridium complexes with bidentate,^31^ pincer ligands,^32^ and rhodium analogues have been tested^33^ NHCs have still proven to reflect the best ligand class and they have been modified to work in aqueous solution.^34^ In fact SABRE transfers its hyperpolarisation successfully through multiple bonds to nitrogen containing compounds such as nicotinamide,^35^ isoniazid,^36^ pyrazole,^37^ acetonitrile^38^ and amines^39^ to improve the detectability of appropriate ^1^H, ^13^C, ^31^P, ^15^N and ^19^F responses.^40^–^44^ It has also proven possible to use radio frequency excitation to mimic the low-field condition in order to allow high-field transfer.^15^,^45^,^46^ A further exciting opportunity revealed by SABRE is reflected in its ability to create hyperpolarised magnetic states with long lifetimes,^47^ an array of research that is of growing importance as hyperpolarisation methods are beginning to feature in clinical diagnosis.

One of the limitations of SABRE has proven to be associated with its ability to hyperpolarise sterically encumbered agents due to the steric properties of the commonly used NHC IMes complex which prevents adequate binding.^48^ However, without some steric interaction ligand loss will be slow.^26^ Here we prepare a series of complexes using a range of asymmetrically substituted NHC's where we maintain some steric congestion and assess them for their SABRE activity. We achieve this by maintaining one mesityl arm in conjunction with the introduction of a smaller substituent to the second nitrogen centre. These substituents were selected carefully to prevent catalyst deactivation through well-known C-H bond activation.^49^ The use of asymmetry in this way to drive exchange is somewhat analogous to the ansa-bridged metallocenes that are used to create isotactic polymers.^50^ We start our studies with pyridine, which reflects the most widely used model ligand in SABRE^51^ before considering 3,4- and 3,5-lutidine so as to introduce additional steric interactions that we predict will lead to improved SABRE behaviour. It is noteworthy that previous studies with lutidine have identified it as a promising tool for pH imaging although steric effects acted to limit SABRE-hyperpolarization effects^52^,^53^ and quench it totally in *ortho*-substituted lutidines and picolines.^54^

## Results and discussion

**Scheme 3:**
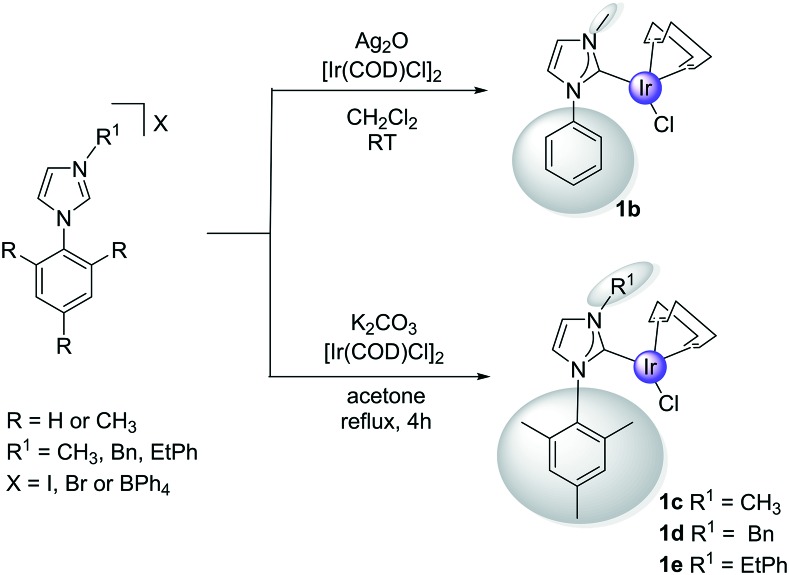
Synthetic routes to **1** containing the specified *C*_2_-asymmetric carbene.

**1a** and the asymmetric complexes **1b–1e** were prepared according to Scheme 3. In the case of the yellow **1b**, this necessitated the silver(i) oxide mediated transmetalation of [Ir(COD)Cl]_2_. However, for **1c–1e** a modified literature procedure^55^ involving BPh_4_^–^ salts, [Ir(COD)Cl]_2_ and K_2_CO_3_ was employed. In all cases the yields exceeded 60% and the products were characterised by 2D NMR spectroscopy, mass spectrometry and elemental analysis as detailed in the ESI.† X-ray data for **1e** is included which map on to other reported structures.

### SABRE activity analysis of precatalysts **1a–1e**

#### (a) Pyridine SABRE activity

**Scheme 4:**
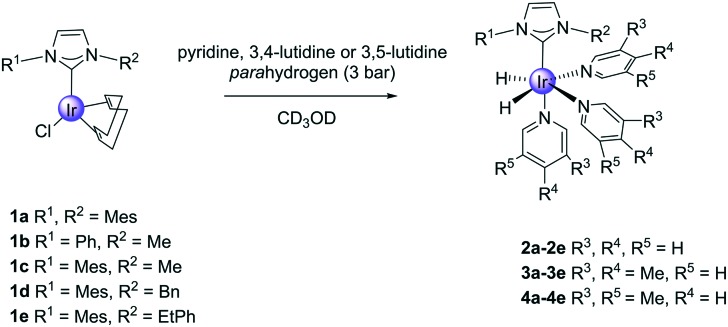
Formation of products **2**, **3** and **4** from reactions with **1**.

The reactivity of precatalysts **1** towards pyridine and H_2_ was first investigated. In order to do this, each of the complexes **1** were dissolved in CD_3_OD solutions that contained pyridine (py, 5-fold excess) prior to adding *p*-H_2_. In all cases, the solutions changed colour from yellow to colourless as **2** forms, with generic formula [Ir(*L*_NHC_)(H)_2_(py)_3_]Cl, where *L*_NHC_ is the corresponding monodentate carbene of Scheme 4. Hydride resonances were readily seen at around –22 ppm in these NMR spectra due to these species. We note that when **1b** was re-examined with ^15^N labelled pyridine the corresponding hydride signal exhibits a ^2^*J*_H–(15)N_ = 19.6 Hz splitting; two ^15^N resonances can be seen at 236.1 and 253.4 ppm for the corresponding axial and equatorial ligands respectively.

The SABRE activities of these species were then compared at 298 K through a series of ^1^H NMR studies using the ‘shake and drop’ method (see ESI†). The resulting per proton *ortho* phenyl ^1^H NMR resonance signal gains, and the total free pyridine substrates ^1^H NMR signal gains, are reported in Table 1 for observations at 9.4 T. It can be readily seen that the order of SABRE efficiency is **2a** > **2d** > **2e** > **2c** > **2b** for both data types. These values suggest that the performance of none of these new complexes compare favourably with that of **2a** for pyridine signal enhancement. In order to identify the reason for this, the corresponding pyridine ligand loss rates were determined. These are detailed in Table 1 and follow the same trend. Hence we can conclude that the associated ligand loss rates are all too small, when compared to **2a**, for optimal behaviour. This observation is consistent with reported literature behaviour.^56^,^57^ When these experiments were repeated at 313 K, the corresponding signal gains increased although **2b** proved unstable (see ESI†) and is not discussed further. Notably, the overall SABRE efficiency trend changes to **2a** > **2d** > **2c** > **2e** at 313 K.

**Table 1:** Rate of ligand loss per molecule in solution, level of pyridine ^1^H NMR signal enhancement (fold) at 9.4 T and 298 K and 313 K for **2**

Complex	**2a**	**2b**	**2c**	**2d**	**2e**
Pyridine ligand loss rate (298 K, s^–1^)	13.35	—	0.51	0.71	1.28
*Ortho* pyridine proton signal gain (per proton) and total proton gain, 298 K (fold)	–1452 ± 81	–0.77 ± 0.04	–48 ± 2	–95 ± 5	–68 ± 3
	5246 ± 262	2.1 ± 0.1	147 ± 7	304 ± 15	209 ± 10
Pyridine ligand loss rate (313 K, s^–1^)	87.82	—	3.66	6.82	7.36
*Ortho* pyridine proton signal gain (per proton) and total proton gain, 313 K (fold)	–414 ± 21	—	–158 ± 8	–196 ± 10	–130 ± 6
	3137 ± 156	—	544 ± 27	677 ± 34	450 ± 22

#### (b) 3,4-Lutidine and 3,5-lutidine SABRE activity

**Fig. 1:**
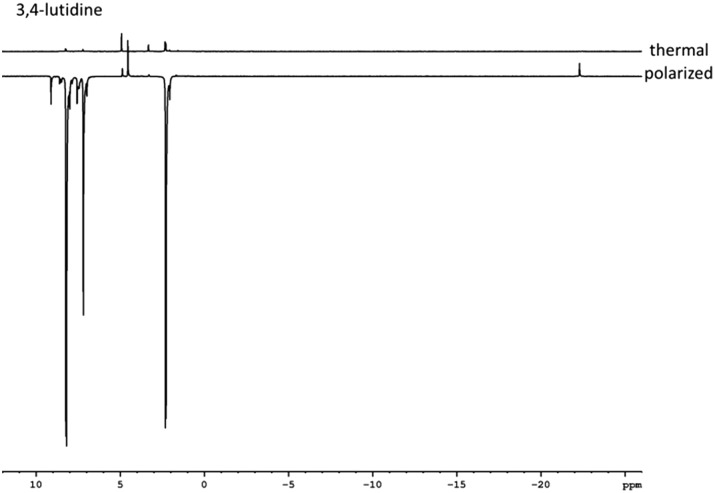
Typically thermal and hyperpolarised ^1^H NMR spectra of 3,4-lutidine achieved by **3d** illustrating the hyperpolarisation effect.

In view of the fact that pyridine binds too tightly in **2b–2e** we tested the bulkier 3,4- and 3,5-lutidine. These targets have p*K*_a_ values of 6.28 and 5.85 (ref. 58) respectively compared to 5.3 for pyridine.^59^ Hence they should bind more strongly if the process is not sterically inhibited and their ligand loss rates should be lower. When CD_3_OD solutions of **1** and 5-equivalents of these lutidines were examined under 3 bar of *p*-H_2_, the formation of [Ir(H)_2_(lut)_3_(*L*_NHC_)]Cl (**3** and **4**) was indicated as detailed in the ESI† in all cases. Tables 2 and 3 summarise the hydride chemical shifts of **3** and **4**, the associated slower lutidine ligand loss rate and the *ortho* proton and total proton signal enhancement values that were obtained after SABRE transfer at 60 G and detection at 9.4 T (Fig. 1).

**Table 2:** Hydride peak positions, 3,4-lutidine loss rate, and indicated ^1^H NMR signal enhancements yield for 3,4-lutidine after SABRE at 298 K

Complex	**3a**	**3b**	**3c**	**3d**	**3e**
Ir–H chemical shift (ppm)	–22.80	–22.42	–22.09	–22.27	–22.32
Ligand loss rate (s^–1^)	9.72	—	0.39	0.76	1.9
*Ortho* proton signal gain (per proton) and total proton gain (fold)	–270 ± 13	—	–69 ± 3	–86 ± 4	–33 ± 2
	1709 ± 85	—	471 ± 23	506 ± 25	223 ± 11

**Table 3:** Hydride peak positions, 3,5-lutidine loss rate, and ^1^H NMR signal enhancements for 3,5-lutidine after SABRE at 298 K

Complex	**4a**	**4b**	**4c**	**4d**	**4e**
Ir–H chemical shift (ppm)	–22.79	–22.24	–22.35	–22.36	–22.34
Ligand loss rate (s^–1^)	2.10	0.34	1.01	2.14	1.9
*Ortho* proton signal gain (per proton) and total proton gain (fold)	–145 ± 7	–1.7 ± 0.1	–125 ± 6	–258 ± 13	–86.7 ± 4
	740 ± 37	10.5 ± 0.5	719 ± 36	1615 ± 81	504 ± 25

The signal enhancements seen for aromatic ^1^H resonances of 3,4-lutidine with **3b–3e** at 298 K proved to be larger than those seen for pyridine with **2b–2e**. This suggests that the result of the chemical structure change and which focuses polarisation transfer into three rather than five proton sites, as for pyridine, is beneficial; this view is consistent with the comparatively poor methyl proton enhancements seen. Upon warming to 313 K the resulting signal gains increased such that **3a** yields an average aromatic proton signal gain of 1700-fold whilst **3d** remains the best competitor with a value of 510-fold.

In contrast, the bulkier 3,5-lutidine shows much better signal enhancements at 298 K than 3,4-lutidine, which is now reflected by the total gain of 1615-fold with **4d**. In both cases, the NHC 1-mesityl-3-benzylimidazole yields the best catalyst (**3d** and **4d**) and we note there are two pairs of *ortho* phenyl protons in [Ir(H)_2_(lut)_3_(*L*_NHC_)]Cl that provides a ^4^*J*_HH_ coupling link to their hydride ligands which should be similar to those for pyridine. It is not surprising that both of these positions therefore receive strong polarisation under similar SABRE. Furthermore, it can be seen that, as predicted, the ability of the SABRE target to receive hyperpolarisation is controlled by the ligand scaffold.

##### Continuous flow experiments

The variation in the level of SABRE observed is therefore strongly dependent on the catalyst and substrate combination. According to the literature, it is also influenced by the magnetic field experienced by the sample at the point of polarisation transfer. This situation arises because polarisation transfer is relayed *via* the scalar coupling in the catalyst when under weak-field conditions. Hence the chemical shift difference that exists between the *para*hydrogen-derived hydride ligands and the interacting spin-½ nuclei of the ligated substrate influence the level of polarisation transfer observed.^12^

**Fig. 2:**
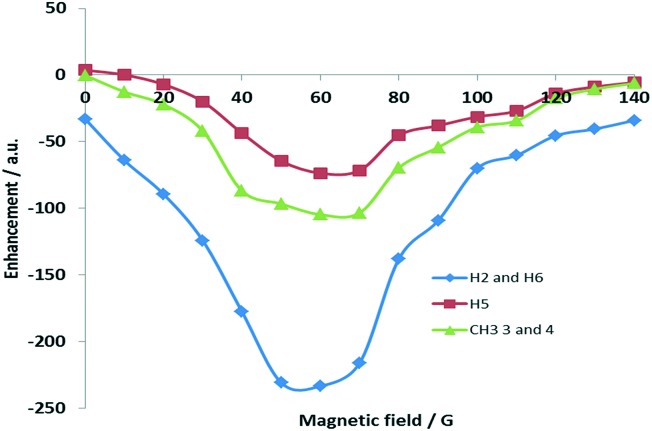
Polarisation transfer field profile of 3,4-lutidine using precursor **1d**.

We used an automated polarizer that has been described previously^60^,^61^ to examine this effect by reference to pyridine and 3,4-lutidine. Fig. 2 shows how the ^1^H NMR signal gain for 3,4-lutidine varies with the changing polarisation transfer field when using catalyst **3d**. For ^1^H transfer, the optimal value for pyridine was 70 G whilst for the aromatic resonances of 3,4-lutidine it is 60 G. It appears, however, that optimal transfer into the aliphatic groups is more efficient at 70 G. It is highly noteworthy that the methyl resonances of 3,4-lutidine are substantially enhanced when compared to related reports.^35^

**Fig. 3:**
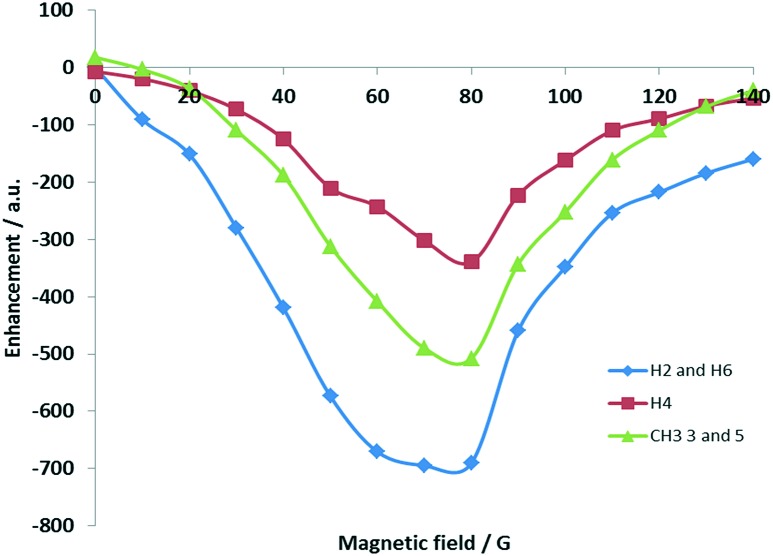
Polarisation transfer field profile for 3,5-lutidine based on precursor **1d**.

Fig. 3 shows the corresponding polarisation transfer field profile for 3,5-lutidine achieved with **4d**. The optimal field value for polarisation transfer now lies between 70 and 80 G with the methyl groups receiving a far greater share of the signal.

##### Effects of NMR relaxation

In order to further rationalise this behaviour, the relaxation times of the protons of these three substrates were measured at 9.4 T in the absence of catalysts in methanol-*d*_4_ whilst under H_2_ as detailed in Table 4. The corresponding values determined under SABRE conditions are also detailed. It is clear that, as expected, the presence of the catalyst consistently reduces these values.^35^,^62^ We therefore determined the corresponding relaxation times of key protons in **3** and **4** when they were prepared from **1** with just a 3.05 fold excess based on iridium.

**Table 4:** Observed *T*_1_ values for the indicated protons of 3,4-lutidine and 3,5-lutidine in the free material at 9.4 T without, and in the presence of the indicated catalyst, alongside the corresponding values when bound as a ligand *trans* to hydride. The initial ratio of **1** to the indicated ligand was 1 : 3.05 to ensure free substrate was present

Sample/agent	*T*_1_ (/s) value (site)		
3,4-Lutidine	*Ortho*	*Meta*	CH_3_
Without	5.75 & 5.58	5.14	3.31 & 3.83
**3a**, free ligand	2.83 & 2.14	6.45	1.80 & 1.82
**3a**, coordinated	3.20 & 2.49	5.09	1.51 & 1.20
3,5-Lutidine			
Without	21.30	19.58	6.63
**4a**, free ligand	3.23	6.26	2.10
**4a**, coordinated	3.13	4.30	2.44
**4c**, free ligand	3.86	5.47	2.45
**4c**, coordinated	2.97	6.91	1.92
**4d**, free ligand	3.74	6.63	2.49
**4d**, coordinated	3.81	6.34	2.35

From all these data it can be seen that whilst **4d** has a very similar ligand loss rate to **4a**, the total signal enhancement it provides is improved by almost 50%. This change is clearly due to less efficient relaxation within the catalyst, which improves by 18% in **4d***versus***4a**. In support of this, **4c** and **4a** actually also deliver similar signal gains with similar relaxation times even though their ligand exchange rates differ by >50%. In view of these observations we can state that the effects of ligand exchange on SABRE activity must be less critical than those of relaxation within the catalyst.

### ^13^C SABRE transfer with **3** and **4**

It has also been shown that SABRE can enhance the ^13^C responses of molecules. When the ^13^C hyperpolarisation of pyridine was evaluated for **2a**, limited ^13^C signal gains are observed even though the IMes derivate works extremely well for ^1^H. This suggests that the ligand exchange rate is too fast (13.35 s^–1^) for substantial direct ^13^C transfer at this field. In contrast, the results with slower exchanging **2b–2e** prove better with **2d** being the most efficient. The *ortho* and *meta*^13^C positions exhibit the strongest signal gains on the molecule. When the polarisation transfer field (PTF) dependence was considered, optimum transfer was achieved at 50 G for **2d** with the total carbon signal enhancement being 430-fold at this value.

**Fig. 4:**
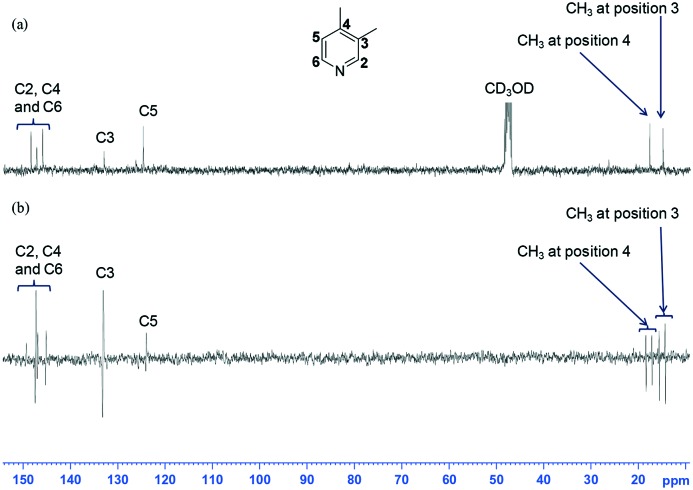
(a) ^13^C{^1^H} NMR spectrum recorded with 1024 scans for a mixture of **3d** and 3,4-lutidine without *p*-H_2_; (b) ^13^C NMR spectra recorded in one scan on the same sample after SABRE showing the corresponding hyperpolarised carbon signals (the vertical expansion setting used to display the spectrum in Fig 4 (a) is four times that used in Fig. 4 (b)); the relaxation delay was 3 seconds alongside a 30° excitation pulse.

When 3,4-lutidine was examined, **3d** and **3e** proved to be similarly effective. Fig. 4 shows a typical NMR spectrum that was obtained with **3d**. For context, when a normal ^13^C{^1^H} NMR spectrum was acquired, 1024 scans were needed to see the associated carbon signals (Fig. 4a).

The signals that are seen in these fully coupled NMR spectra contain anti-phase character that is associated with the detection of a heteronuclear longitudinal two spin order term (*I*_z_*S*_z_). In each case, the anti-phase splitting is ∼8 Hz in size and reflects a long-range rather than direct one bond CH splitting. This is fully consistent with related reported observations.^15^,^38^ The SABRE ^13^C activity of **3d** was also investigated more rigorously using the flow apparatus. The two inequivalent *meta*- and *para*-methyl ^13^C signals (positions 3 and 4) of 3,4-lutidine were found to show relatively large enhancements after transfer between 0–90 G, with no signal seen after transfer between 90–140 G. The *meta*-methyl ^13^C signals (position 3) generally exhibited lower polarisation levels to that of the *para*-methyl resonance (position 4). In the case of the aromatic region, the *meta* quaternary carbon at position 4 on 3,4-lutidine showed an individual enhancement value of 544-fold at 40 G whereas the *ortho*-**C**H value was 560-fold. The *meta*-**C**H resonance showed minimal polarisation enhancement at 40 G and this increase to 50-fold at 30 G. The trend in signal intensity with polarisation transfer field for these ^13^C resonances is very different to that seen in the corresponding ^1^H NMR spectra.

**Fig. 5:**
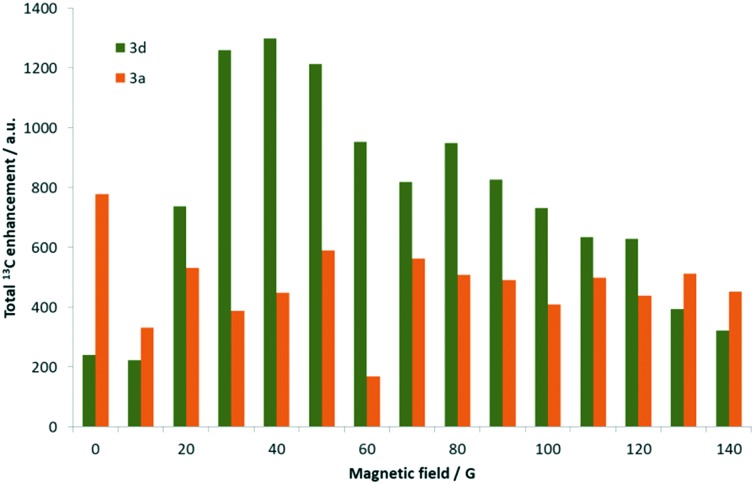
Graph comparing the total ^13^C enhancement observed for the carbon resonances of 3,4-lutidine when using complex **3d** (green) and **3a** (orange) respectively as a function of the strength of the polarisation transfer field.

In addition, the maximum signal gain seen in the ^1^H NMR spectra is reflected in the *ortho*-C**H** resonances, followed by the methyl groups, and finally the *meta*-C**H** proton. Comparatively, **3a** still yields significantly lower ^13^C polarisation than **3d**, achieving only a 222-fold gain for the *meta*-quaternary carbon at 70 G. While the *ortho*-CH signal could be seen, the remaining carbon resonances exhibited signal enhancements of 20–300 fold depending on the magnetic field strength tested. Hence, the total ^13^C signal enhancements seen for **3d** were 1300-fold after transfer at 40 G, which was significantly higher than the corresponding 780-fold at 0.5 G achieved with **3a** as detailed in Fig. 5.

**Fig. 6:**
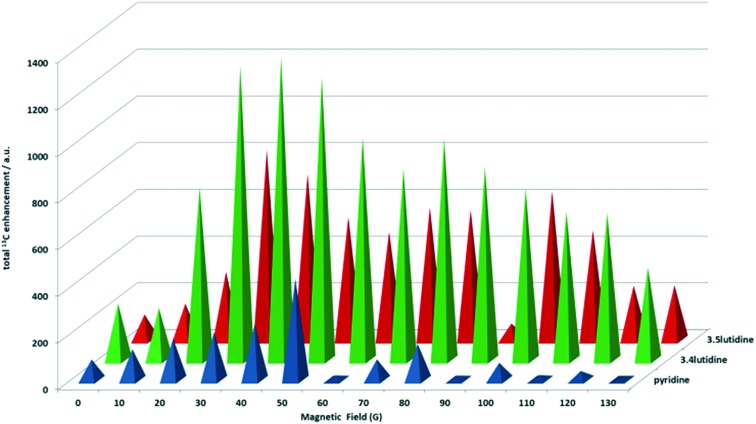
Graph comparing the ^13^C enhancement observed for the carbon resonances of pyridine, 3,4-lutidine and 3,5-lutidine when using complex **3d** as a function of the strength of the polarisation transfer field.

Precursor **1d** also achieved the optimal ^13^C NMR signal enhancement in 3,5-lutidine and the results showed a lower level of enhancement compared to 3,4-lutidine, which is in agreement with the corresponding ^1^H NMR changes. Now signals for all of the carbon sites, except the *para* position, are clearly visible after transfer in fields between 0 and 130 G. At 30 G, the optimum magnetic field, the total carbon NMR signal gain was 800-fold, with a 380-fold contribution coming from the quaternary *meta* carbon position, 96-fold for the methyl carbons, and the remainder being due to *ortho*^13^C signal. The full PTF profile is presented in Fig. 6.

### Early reaction stages

During such SABRE studies it has proven common in the early stages of reaction to observe complexes of the type [Ir(H)_2_(COD)(NHC)(sub)]Cl. These materials form transiently as **1** converts into the final tris substituted product. They actually reflect H_2_ addition to the initial substitution product [Ir(sub)(NHC)(COD)]Cl which forms from the precursor [Ir(NHC)(COD)Cl]. Their observation is important because If the initial substitution is slow then inefficient H_2_ addition to [Ir(NHC)(COD)Cl] occurs. However, while the resulting product [Ir(H)_2_(NHC)(COD)Cl] goes on to yield common [Ir(H)_2_(sub)(NHC)(COD)]Cl the reaction can take weeks at 298 K. For completeness, the COD ligand present in these complexes is converted to COE and finally COA during this process.

We therefore examined the early stages of these reactions with pyridine but observed [Ir(py)(NHC)(COD)]Cl to form only when the NHC was IMes. When the associated sample of **1b** containing a 5-fold excess of pyridine was exposed to *para*hydrogen in methanol-*d*_4_ at 263 K, and the solution warmed to 273 K, no reaction was initially evident in the corresponding 1-scan ^1^H NMR spectrum. However, when 16-scans are averaged, a pair of very weak antiphase hydride signals is detected at –12.68 and –17.67 ppm that share a common *J*_H–H_ coupling of –10 Hz. Subsequently the species giving rise to this material converts into **2b**, although significant decomposition is evident.

**Fig. 7:**
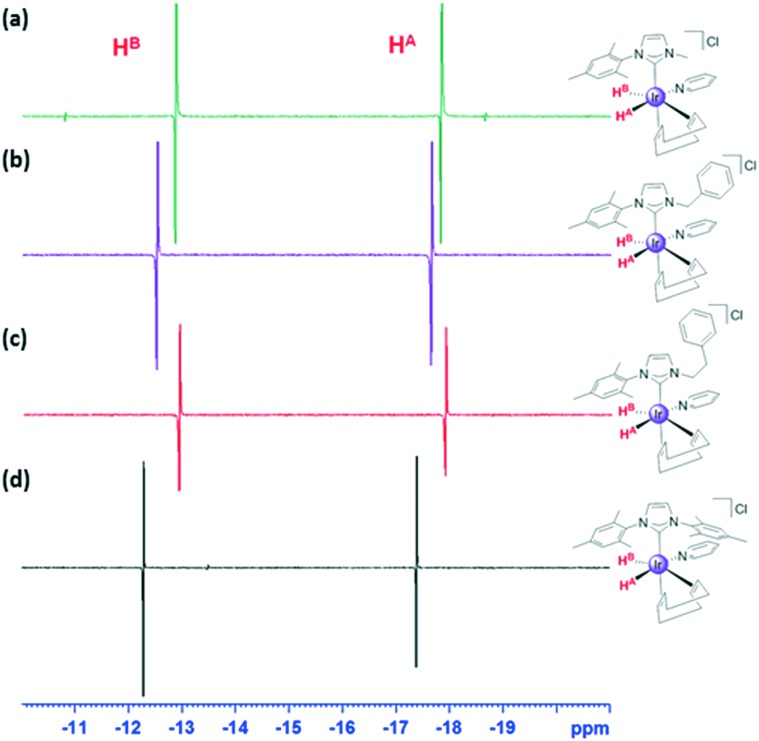
Hydride region of a series of ^1^H NMR spectra recorded using a π/4 pulse (400 MHz, CD_3_OD, 273 K) showing signals for: (a) **5c**; (b) **5d**; (c) **5e** and (d) **5a**.

When a similar sample of **1c** was exposed to *p*-H_2_ at 273 K in methanol-*d*_4_ a pair of similar strongly enhanced antiphase hydride signals is detected. They now occur at –12.86 and –17.82 ppm and share a *J*_HH_ coupling of –5.5 Hz. Similar observations result from **1d** and **1e** and the associated NMR spectra are illustrated in Fig. 7. We note that the rate of formation of final products **2d** and **2e** exceeds that of **2b** and **2c**.

## Experimental

The asymmetric monocarbene ligands of Scheme 1 were synthesised by modifying previously published procedures.^63^ Phenylimidazole, or mesitylimidazole, were reacted with the respective iodomethane, benzyl bromide or phenethyl bromide in acetonitrile and stirred at 25 °C or at reflux. The solvent was evaporated and crude brown-oil was recrystallised from methanol : diethyl ether mixtures to give off-white solids [PhIMe]I, [MesIMe]I, [MesIBn]Br or [MesIEtPh]Br. These ligands were dissolved in dichloromethane and stirred for 1 hour with NaBPh_4_ to exchange the counter-ion. The mixture was filtered and the solvent evaporated *in vacuo* to yield [MesIMe]BPh_4_, [MesIBn]BPh_4_ or [MesIEtPh]BPh_4_ as white crystalline solids.

### Synthesis of Ir(i) complexes containing asymmetric monocarbene ligands **1b–1e**

**1b** was synthesised by the silver(i) transmetallation of [Ir(COD)Cl]_2_. The reaction mixture was stirred overnight at 25 °C, filtered and the solvent evaporated. The crude product was then dissolved in dichloromethane, filtered, isolated and recrystallised from dichloromethane: hexane as a bright yellow solid. **1c–1e** were synthesised by heating [Ir(COD)Cl]_2_ and K_2_CO_3_ in acetone with the corresponding ligand at reflux for 4 hours. After cooling, the mixture was filtered, and the solvent evaporated. The crude product was redissolved in dichloromethane, and a white solid precipitates. [Ir(COD)(MesIMe)Cl] (**1c**), [Ir(COD)(MesIBn)Cl] (**1d**) and [Ir(COD)(MesIEtPh)Cl] (**1e**) resulted as yellow solids, and were used without further purification. All these complexes were air-stable as solids.

Single crystals of and **1e** suitable for X-ray structure determination were obtained by slow diffusion of a concentrated dichloromethane solution of the complex in hexane at –20 °C (see ESI†).

## Conclusions

The results presented in this work demonstrate that the asymmetric carbene ligands of Scheme 2 can be used to prepare complexes of the type [Ir(NHC)(COD)Cl] (**1**). These complexes have been shown to react with hydrogen and substrates pyridine, 3,4-lutidine or 3,5-lutidine to form the corresponding Ir(iii) products [Ir(H)_2_(NHC)(sub)_3_]Cl (**2–4**) of Scheme 3. These products were then assessed for activity in the SABRE process, which involves the catalytic transfer of polarisation from *p*-H_2_ into the substrate to enhance its NMR detectability. Key parameters in this process, namely the ligand exchange rates, and the relaxation times of the associated NMR resonances have been assessed and compared to those of the symmetric carbene bearing [Ir(H)_2_(IMes)(sub)_3_]Cl (**2–4a**), the established SABRE reference catalyst. For pyridine and 3,4-lutidine poor activity was observed as a consequence of slow ligand loss due to the reduced steric bulk associated with these NHC's relative to IMes and strong interactions with the associated nitrogen centres due to their high basicity. However, when 3,5-lutidine was examined, the enhanced steric encumbrance of this substrate out ways the p*K*_a_ benefit and increases the rates of ligand loss for the asymmetric NHC's such that they become competitive with their IMes derivatives. The result of this process is that **4d**, achieved an *ortho* proton signal gain of 258-fold per proton in 3,5-lutidine in contrast to the 145-fold value achieved by **4a** at 9.4 T.

One further benefit of these catalysts is reflected in their ability to sensitise the ^13^C response of these agents. Now the enhanced stability works favourably as evidenced by the spectra of Fig. 4. It is therefore likely that asymmetric carbenes offer a route to further improve the ability of SABRE to enhance the MR response of sterically encumbered substrates and hence the range of applications. For efficient polarization transfer to ^13^C, the utilization of a micro-Tesla field in conjunction with the presence of ^15^N to relay polarization transfer has proven highly efficient thereby suggesting a route to improve on these results.^64^,^65^

## Conflicts of interest

There are no conflicts to declare.

## Supplementary Material

Supplementary information

Crystal structure data
