# Supplementary material for: Harnessing asymmetric N-heterocyclic carbene ligands to optimise SABRE hyperpolarisation
Source: Catal Sci Technol. 2018 Sep 3;8(19):4925–33. doi: 10.1039/c8cy01214h (PMC6171487; doi:10.1039/c8cy01214h)
Supplement: Supplementary file 1 [file CY-008-C8CY01214H-s001.pdf]

Electronic Supplementary Material (ESI) for Catalysis Science & Technology.
This journal is © The Royal Society of Chemistry 2018

Supporting Information for

Harnessing asymmetric N-heterocyclic carbene ligands to optimise SABRE hyperpolarisation

Chin Min Wong,[a,b] Marianna Fekete,[c] Rhianna Nelson-Forde,[c] Mark R. D. Gatus,[b] Peter J. Rayner,[c] Adrian C. Whitwood,[c] Simon B. Duckett,*[c] and Barbara A. Messerle.*[b]

[a.] *School of Chemistry, University of New South Wales, Sydney 2052, Australia.*

[b.] *Department of Molecular Sciences, Macquarie University, North Ryde 2109, Australia*

[c.] *Centre for Hyperpolarisation in Magnetic Resonance, York Science Park, University of York, Heslington, York YO10 5NY, United Kingdom*

** email: simon.duckett@york.ac.uk, barbara.messerle@mq.edu.au*

Table of contents:

Harnessing asymmetric carbenes to optimise SABRE hyperpolarisation 1

General Experimental Procedures and Characterisation Data 3

General Procedure for preparation of NMR samples for SABRE analysis 4

General calculation for ^{1}H NMR enhancement factors..5

General calculation for ^{13}C NMR enhancement factors 5

Synthesis of Complexes 9

Characterisation of active complexes in the presence of pyridine 13

Characterisation of the active complex formed in the presence of 3,4-lutidine......................16

Characterisation of the active complex formed in the presence of 3,5-lutidine......................19

Ligand exchange rates and proton NMR signal enhancement..24

SABRE experiments, using Flow-apparatus...26

^{13}C NMR enhancements data 31

X-Ray analysis of 1e 34

References 46

General Experimental Procedures and Characterisation Data

Unless otherwise stated, all manipulations were carried out under inert atmosphere using standard Schlenk techniques or in a nitrogen or argon-filled Braun glovebox. All reagents were purchased from Aldrich Chemical Company Inc. or Alfa Aesar Inc. and used as received unless otherwise noted. $IrCl_3.xH_2O$ was purchased from Precious Metals Online PMO P/L. For the purposes of air sensitive manipulations and in the preparation of air sensitive metal complexes dichloromethane, acetonitrile, diethyl ether and pentane were dispensed from a PuraSolv solvent purification system. Methanol and hexane were used as received. The bulk compressed gases argon (>99.999%) were obtained from Air Liquide and used as received. Nitrogen gas for Schlenk line operation comes from in-house liquid nitrogen boil-off. $[Ir(COD)Cl]_2$,[1] 1-phenyl-1*H*-imidazole,[2] 1-mesityl-1*H*-imidazole,[3] 3-methyl-1-phenyl-1H-imidazol-3-ium iodide,[4] 1-mesityl-3-methyl-1H-imidazol-3-ium iodide,[4] 1-mesityl-3-methyl-1H-imidazol-3-ium tetraphenylborate[5] and [Ir(IMes)CODCl] (**1a**)[6] were synthesized using literature procedures. *Para*hydrogen (*p*-H_2) was prepared by cooling dihydrogen over Fe_2O_3 at 28K and introduced to the NMR tube using a home-built generator. For the flow measurements, *para*hydrogen was supplied using a Bruker *para*hydrogen generator.

^1H and $^{13}C\{^1H\}$ NMR spectra were recorded on Bruker Avance III series spectrometers operating at 400, 500 and 600 MHz (^1H) and 100, 125 and 150 MHz (^{13}C) and 50.7 MHz (^{15}N) respectively. ^1H and ^{13}C NMR chemical shifts were referenced internally to residual solvent resonances. ^{15}N NMR chemical shifts were referenced externally to ^{15}N-urea. Unless otherwise stated, spectra were recorded at 298 K and chemical shifts (δ), with uncertainties ± 0.01 Hz for ^1H and ± 0.05 Hz for ^{13}C and ^{15}N, are quoted in parts per million, ppm. Coupling constants (J) are quoted in Hz and have uncertainties of ± 0.05 Hz for ^1H-^1H and ^1H-^{15}N and ± 0.5 Hz for ^{13}C-^{11}B and ^{15}N-^{15}N. Deuterated solvents were purchased from Cambridge Stable Isotopes or Sigma Aldrich and used as received. Air sensitive NMR samples were prepared in an inert gas glovebox or by vacuum transfer of deuterated solvents into NMR tubes fitted with a Young's teflon valve. For air sensitive NMR samples, dichloromethane-d_2 and chloroform-d were distilled over calcium hydride. Microanalyses were carried out at the at the Campbell Micro-analytical Laboratory, University of Otago, New Zealand or at the Research School of Chemistry, The Australian National University, Canberra, Australia or at the Chemical Analysis Facility, Department of Chemistry and Biomolecular Sciences,

Macquarie University, Sydney, Australia.. Mass spectra were acquired using a Thermo LTQ Orbitrap XL located in the Bioanalytical Mass Spectrometry Facility (BMSF) in UNSW. M is defined as the molecular weight of the compound of interest or cationic fragment for cationic metal complexes.

General Procedure for preparation of NMR samples for SABRE analysis

SABRE experiments were conducted either in a 5 mm NMR tubes fitted with Young's valves (Method 1) or an automated polarizer (Method 2).

Method 1

NMR samples were prepared in 5 mm NMR tubes fitted with Young's valves. Samples were degassed three times on a high vacuum Schlenk line whilst immersing the solution in a dry CO_2/acetone slush bath prior to *p*-H_2 (3 bar) addition. Typical procedures for reactions with pyridine, 3,4-lutidine and 3,5-lutidine are described. NMR characterisation data was collected using a range of 1D and 2D methods that include nOe, COSY and HMQC procedures. In a typical experiment, the iridium complex, [Ir(NHC)CODCl] (**1a**-**1e**) (5 mM), and five equivalents of either pyridine, 3,4-lutidine or 3,5-lutidine (25 mM) were dissolved in methanol-d_4 (0.6 ml). The bright yellow mixture was degassed and *para*hydrogen at a pressure of 3 bar was added. When the samples were analysed at 298K, samples were then shaken for 10 s at 65 G of the NMR spectrometer before rapidly transported into the magnet for NMR measurements. When the samples were analysed at 313K, the samples were first placed in a water bath at 313K for 3 minutes. The samples were then quickly shaken for 10 s at 65G of the NMR spectrometer before rapidly transported into the magnet, which was previously heated to 313K for NMR measurements.

Method 2

The automated polarizer method was used to analyse a mixture containing [Ir(NHC)CODCl] (**1a**, **1d**, **1e**) (5 mM) and 3,4-lutidine or 3,5-lutidine (25 mM) in methanol-d_4 (3 mL) at 313K. The solvent, catalyst and substrate were placed in a glass enclosed tube with two side arms (mixing chamber). The mixing chamber was placed in a tuneable copper coil (0-140 G) situated in a magnetic field, All magnitudes of the magnetic field in which polarization transfer occurs are stated without correction for this local field. The system is entirely

automated - the liquid and gas flow from the mixing chamber are computer controlled *via* the pulse program. The mixing chamber was heated within by an external circulating heated water pump to 313K. *Para*hydrogen was introduced continuously through an external *para*hydrogen generator into the mixing chamber to activate the catalyst. Nitrogen gas was used to transfer the hyperpolarized solution from the mixing chamber to the NMR probe head for measurement. Before taking a measurement ^{1}H NMR measurement, the *para*hydrogen bubbling time into the mixture was set to be 10s. Before taking a measurement ^{13}C NMR measurement, the *para*hydrogen bubbling time into the mixture was set to be 30s. The transportation time was calibrated to 0.4 s. A further delay of 0.5 s was allowed for settling of the sample prior to signal acquisition.

General calculation for ^{1}H NMR enhancement factors

The ^{1}H NMR signal enhancement was calculated by using the following equation:

$$signal\ enhancement = \frac{signal\ of\ polarized\ sample\ measured\ by\ integral}{signal\ of\ unpolarized\ sample\ measured\ by\ integral}$$

The reference spectrum, or the spectrum of the unpolarised sample was measured using the same hyperpolarised sample, were recorded after it had fully relaxed. The reference spectrum and the hyperpolarized spectrum were measured using the same acquisition, delay and receiver gain parameters. The raw integrals of the relevant resonances in the reference and the hyperpolarized spectra were used to determine the enhancement levels using the equation above. The results are not corrected for relaxation losses during the transfer time (0.9 s for flow measurements and *ca.* 4 s for NMR tube measurements) into the NMR magnet and hence reflect experimentally observed values.

General calculation for ^{13}C NMR enhancement factors

^{13}C enhancements were calculated by taking the raw integral of the $^{13}CD_3OD$ peak observed from the solvent in the sample after equilibration inside the magnet for 1 minute. $^{13}CD_3OD$ was present in each sample at a concentration of 24.6 M and the resulting SABRE hyperpolarized signal was then scaled according to the concentration of substrate in solution relative to the methanol signal to give the final enhancement value for ^{13}C.[7]

Synthesis of ligands

Synthesis of MesIBn.Br

Mesityl imidazole (250 mg, 1.34 mmol) was dissolved in acetonitrile (20 mL). Benzyl bromide (248 mg, 1.45 mmol) was then added dropwise to the brown mixture. The mixture was stirred and heated at reflux overnight. The brown mixture was cooled to room temperature and the solvent was evaporated to give a dark brown oily residue. The crude product was dissolved in a small amount of methanol (3 mL) and diethyl ethyl (30 mL) was added to the mixture with stirring to give the product as a fluffy off-white solid.

Yield: 407 mg, 85%

^{1}H NMR ($(CD_3)_2SO$, 600 MHz, 298K): δ 9.57 (br s, 1H, Im-**H**2), 8.06 (t, $^{3}J_{H\text{-}H}$ = 1.6 Hz, 1H, Im-H4), 7.95 (t, $^{3}J_{H\text{-}H}$ = 1.8 Hz, 1H, Im-H5), 7.44 (m, 5H, *ortho*-C**H**, *meta*-C**H** and *para*-C**H** of phenyl ring), 7.15 (s, 2H, *meta*-C**H** of Mes), 5.53 (s, 2H, C$\mathbf{H}_2$), 2.33 (s, 3H, *para*-C$\mathbf{H}_3$ of Mes), 2.00 (s, 6H, *ortho*-C$\mathbf{H}_3$ of Mes) ppm.

^{13}C{^{1}H} NMR ($(CD_3)_2SO$, 150 MHz, 298K): δ 140.3 (*para*-**C**CH_3 of Mes), 137.6 (Im-**C**2), 134.7 ($\mathbf{C}_q$ of phenyl ring), 134.2 (*ortho*-**C**CH_3 of Mes), 131.1 ($\mathbf{C}_q$ of Mes), 129.3 (*meta*-**C**H of Mes), 129.2 (*ortho*-**C**H of phenyl ring), 128.9 (*meta*-**C**H of phenyl ring), 128.0 (*para*-**C**H of phenyl ring), 124.4 (Im-**C**5), 123.3 (Im-**C**4), 52.4 (**C**H_2), 20.6 (*para*-**C**H_3 of Mes), 16.9 (*ortho*-**C**H_3 of Mes) ppm.

HR-MS (ESI^{+}, MeOH) : *m/z* (%): (100 %) $[M]^{+}$ = $[C_{19}H_{21}N_2]^{+}$ = 277.1693 (Calculated $[M]^{+}$ = 277.1699) amu.

Synthesis of MesIBn.BPh_4

MesIBn.Br (166 mg, 0.464 mmol) was dissolved in dichloromethane (15 mL) and sodium tetraphenylborate (163 mg, 0.476 mmol) was added to the colourless solution. White precipitate immediately forms. The mixture was left to stir for 1 hour and then it was filtered through celite and washed thoroughly with dichloromethane. The solvent was evaporated to give the product as a crystalline off-white fluffy solid.

Yield: 217 mg, 78 %

^{1}H NMR ($CDCl_3$, 600 MHz, 298K): δ 7.39 (br s, 8H, *ortho*-C**H** of BPh_4) overlapped with 7.37 (m, 3H, *ortho*-C**H** and *meta*-C**H** of Ar), 7.04 (s, 2H, *meta*-C**H** of Mes), 6.89 (t, $^{3}J_{H-H}$ = 7.5 Hz, 8H, *meta*-C**H** of BPh_4) overlapped with 6.87 (m, 2H, *para*-C**H** of Ar), 6.81 (t, $^{3}J_{H-H}$ = 7.2 Hz, 4H, *para*-C**H** of BPh_4), 6.04 (t, $^{3}J_{H-H}$ = 1.7 Hz, 1H, Im-**H**4/5), 6.55 (t, $^{3}J_{H-H}$ = 1.6 Hz, 1H, Im-**H**4/5), 5.45 (t, $^{3}J_{H-H}$ = 1.6 Hz, 1H, Im-**H**2), 4.08 (s, 2H, C**H**$_2$), 2.41 (s, 3H, *para*-C**H** of Mes), 1.89 (s, 6H, *ortho*-C**H** of Mes) ppm.

HR-MS (ESI$^+$, MeOH) : *m/z* (%): (100 %) $[M]^+$ = $[C_{19}H_{21}N_2]^+$ = 277.1698 (Calculated $[M]^+$ = 277.1699) amu.

Synthesis of MesIEtPh.Br

Mesityl imidazole (575 mg, 3.62 mmol) was dissolved in acetonitrile (20 mL). 2-Phenylethyl bromide (1000 mg, 5.43 mmol) was then added dropwise to the brown mixture. The mixture was stirred and heated at reflux overnight. The brown mixture was cooled to room temperature and the solvent was evaporated to give a dark brown oily residue. The crude product was dissolved in a small amount of methanol (3 mL) and diethyl ethyl (30 mL) was added to the mixture with stirring to give the product as a fluffy off-white solid.

Yield: 773 mg, 58%

^{1}H NMR (CD_3OD, 400 MHz, 298K): δ 9.20 (t, $^{3}J_{H-H}$ = 1.5 Hz, 1H, Im-**H**2), 8.04 (t, $^{3}J_{H-H}$ = 1.7 Hz, 1H, Im-**H**4), 7.85 (t, $^{3}J_{H-H}$ = 1.8 Hz, 1H, Im-**H**5), 7.29 (apparent t, $^{3}J_{H-H}$ = 7.3 Hz, 2H, *meta*-C**H** of phenyl ring), 7.23 (apparent t, $^{3}J_{H-H}$ = 7.3 Hz, 1H, *para*-C**H** of phenyl ring), 7.20 (apparent d, $^{3}J_{H-H}$ = 7.3 Hz, 2H, *ortho*-C**H** of phenyl ring), 7.10 (s, 2H, *meta*-C**H** of Mes), 4.58 (t, $^{3}J_{H-H}$ = 6.7 Hz, 2H, ImN-C**H**$_2$), 3.24 (t, $^{3}J_{H-H}$ = 6.8 Hz, 2H, Ar-C**H**$_2$),2.30 (s, 3H, *para*-CH_3 of Mes), 1.84 (s, 6H, *ortho*-C**H**$_3$ of Mes) ppm.

^{13}C{^{1}H} NMR (CD_3OD, 100 MHz, 298K): δ 140.2 (*para*-**C**CH_3 of Mes), 137.1 (Im-**C**2), 136.5 (**C**$_q$ of phenyl ring), 134.2 (*ortho*-**C**CH_3 of Mes), 131.0 (**C**$_q$ of Mes), 129.1 (*meta*-**C**H of Mes), 128.7 (*ortho*-**C**H of phenyl ring), 128.6 (*meta*-**C**H of phenyl ring), 126.9 (*para*-**C**H of phenyl ring), 123.9 (Im-**C**5), 123.1 (Im-**C**4), 50.3 (ImN-**C**H_2), 34.9 (Ar-**C**H_2), 20.5 (*para*-**C**H_3 of Mes), 16.6 (*ortho*-**C**H_3 of Mes) ppm.

HR-MS (ESI$^+$, MeOH) : *m/z* (%): (100 %) $[M]^+$ = $[C_{20}H_{23}N_2]^+$ = 291.1854 (Calculated $[M]^+$ = 291.1856) amu.

Synthesis of MesIEtPh.BPh_4

MesIEtPh.Br (146 mg, 0.392 mmol) was dissolved in dichloromethane (15 mL) and sodium tetraphenylborate (137 mg, 0.401 mmol) was added to the colourless solution. White precipitate immediately forms. The mixture was left to stir for 1 hour and then it was filtered through celite and washed thoroughly with dichloromethane. The solvent was evaporated to give the product as a crystalline off-white fluffy solid.

Yield: 222 mg, 93%

^{1}H NMR ($CDCl_3$, 600 MHz, 298K): δ 7.37 (br s, 8H, *ortho*-C**H** of BPh_4), 7.23 (m, 3H, *ortho*-C**H** and *meta*-C**H** of Ar), 6.98 (s, 2H, *meta*-C**H** of Mes), 6.90 (t, $^3J_{H\text{-}H}$ = 7.4 Hz, 8H, *meta*-C**H** of BPh_4), 6.81 (m, 2H, *para*-C**H** of Ar) overlapped with 6.79 (t, $^3J_{H\text{-}H}$ = 7.4 Hz, 4H, *para*-C**H** of BPh_4), 6.51 (br s, 1H, Im-**H**4/5), 6.43 (br s, 1H, Im-**H**4/5), 5.25 (t, $^3J_{H\text{-}H}$ = 1.5 Hz, 1H, Im-**H**2), 3.37 (t, $^3J_{H\text{-}H}$ = 6.3 Hz, 2H, ImN-C**H**$_2$), 2.69 (t, $^3J_{H\text{-}H}$ = 6.3 Hz, 2H, ImN-C**H**$_2$), 2.38 (s, 3H, *para*-C**H** of Mes), 1.68 (s, 6H, *ortho*-C**H** of Mes) ppm.

HR-MS (ESI$^+$, MeOH) : *m/z* (%): (100 %) $[M]^+ = [C_{20}H_{23}N_2]^+$ = 291.1853 (Calculated $[M]^+$ = 291.1856) amu.

Synthesis of Complexes

[Ir(PhIMe)CODCl] **(1b)**

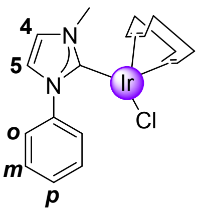

1-methyl-3-phenyl-1*H*-imidazolium iodide (304 mg, 1.07 mmol), Ag_2O (186 mg, 0.802 mmol) and $[Ir(COD)_2Cl]_2$ (374 mg, 0.566 mmol) were added to dichloromethane (20 mL) and the mixture was stirred for 16 h. The reaction mixture was then filtered through celite and the solvent removed from the filtrate (*in vacuo*). The complex [Ir(PhIMe)CODCl] (**1b**) was recrystallised by dissolving the crude solid obtained form the filtrate in the minimum amount of dichloromethane and adding excess *n*-hexane to the mixture until a yellow precipitate was obtained. The precipitate was filtered to yield the desired complex **6** as a yellow solid.

Yield: 368 mg, 0.749 mmol, 70 %.

1H NMR (CDCl3, 400 MHz, 298K): δ 7.99 (m, 2H, *meta*-C**H** of phenyl), 7.49 (m, 2H, *ortho*-C**H** of phenyl), 7.40 (m, 1H, *para*-C**H** of phenyl), 7.13 (d, 1H, Im-**H**5), 6.98 (d, 1H, Im-**H**4), 4.68 (m, 1H, =C**H**-COD), 4.50 (m, 1H, =C**H** of COD), 4.08 (s, 3H, C**H**$_3$), 2.88 (m, 1H, =C**H** of COD), 2.22 (m, 1H, sp^2-COD), 2.15 (m, 2H, , =C**H** of COD), 1.90 (m 1H, C**H**$_2$ of COD), 1.62 (m, 1H, C**H**$_2$ of COD), 1.54 (m, 2H, C**H**$_2$ of COD), 1.35 (m, 1H, C**H**$_2$ of COD), 1.21 (m, 1H, C**H**$_2$ of COD) ppm.

^{13}C{^{1}H} NMR (CDCl$_3$, 100 MHz, 298K): *δ* 180.9 (Im-**C**2), 140.1 (**C**$_q$ of phenyl), 128.8 (*ortho*-**C**H of phenyl), 127.9 (*para*-**C**H of phenyl), 125.3 (*meta*-**C**H of phenyl), 122.5 (Im-**C**4), 121.1 (Im-**C**5), 84.2 (=**C**H of COD), 83.8 (=**C**H of COD), 51.9 (=**C**H of COD), 51.7 (=**C**H of COD), 38.3 (**C**8), 34.2 (**C**H$_2$ of COD), 32.6 (**C**H$_2$ of COD), 29.6 (**C**H$_2$ of COD), 29.5 (**C**H$_2$ of COD) ppm.

Elemental Anal. found: 43.58; H, 4.55; N, 5.56 %; calculated for $C_{18}H_{22}ClIrN_2$: C, 43.76; H, 4.49; N, 5.67 %. ESI-MS (MeOH), *m/z* (%): 457.33 (95) [M-Cl]$^+$ amu.

[Ir(MesIMe)CODCl] (**1c**)

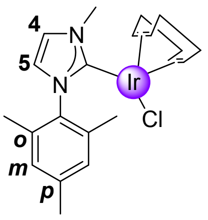

1-mesityl-3-methyl-1*H*-imidazol-3-ium tetraphenylborate (106 mg, 0.203 mmol) and K_2CO_3 (140 mg, 1.02 mmol) were added to acetone (30 mL). After 10 min of stirring the mixture $[Ir(COD)_2Cl]_2$ (69.6 mg, 0.104 mmol) was added, the mixture turned a light orange colour and the mixture was refluxed for 4 h. The solvent was removed in vacuo and the crude mixture redissolved dichloromethane (20 mL) and the mixture was filtered through celite. 80 mL of n-hexane was added to the filtrate and the solvent was reduced to ca. 70 mL, heated to 60 °C and filtered through celite (while hot) to remove any traces of BPh_4 salts. The solvent was removed in vacuo to yield the complex [Ir(MesIMe)CODCl] (**1c**) as a yellow solid.

Yield: 98.1 mg, 0.183 mmol, 90%.

^{1}H NMR: (CD$_2$Cl$_2$, 600 MHz, 298K): δ 7.09 (d, $^3J_{H\text{-}H}$ = 1.7 Hz, 1H, Im-**H**4), 7.05 (br d, 1H, *meta*-C**H** of Mes), 6.96 (br d, 1H, *meta*-C**H** of Mes), 6.81 (d, $^3J_{H\text{-}H}$ = 1.7 Hz, 1H, Im-**H**5), 4.39-4.28 (m, 2H, =C**H** of COD), 4.11 (s, 3H, Im-N-C**H**$_3$), 3.17 (m, 1H, =C**H** of COD), 2.73 (m, 1H, =C**H** of COD), 2.38 (s, 3H, *para*-C**H**$_3$ of Mes), 2.30 (s, 3H, *ortho*-C**H**$_3$ of Mes), 2.25-2.16 (m, 1H, C**H**$_2$ of COD), 2.11-2.01 (m, 1H, C**H**$_2$ of COD), 1.93 (s, 3H, *ortho*-C**H**$_3$ of Mes), 1.85-1.76 (m, 1H, C**H**$_2$ of COD), 1.63-1.55 (m, 2H, C**H**$_2$ of COD), 1.53-1.38 (m, 2H, C**H**$_2$ of COD), 1.25-1.17 (m, 1H, C**H**$_2$ of COD) ppm.

$^{13}C\{^1H\}$ NMR (CD_2Cl_2, 150 MHz, 298K): δ 180.6 (Im-**C**2), 139.0 (*para*-**C**CH_3 of Mes), 137.05 (*ortho*-**C**CH_3 of Mes), 136.3 (*ipso*-**C**$_q$ of Mes), 134.9 (*ortho*-**C**CH_3 of Mes), 129.6 (*meta*-**C**H of Mes), 128.5 (*meta*-**C**H of Mes), 123.1 (Im-**H**5), 122.4 (Im-**H**4), 82.99 (=**C**H of COD), 83.0 (=**C**H of COD), 52.0 (=**C**H of COD), 50.7 (=**C**H of COD), 38.7 (Im-N-**C**H_3), 35.1 (**C**H_2 of COD), 32.7 (**C**H_2 of COD), 30.1 (**C**H_2 of COD), 29.0 (**C**H_2 of COD), 21.2 (*para*-**C**H_3 of Mes), 19.6 (*ortho*-**C**H_3 of Mes), 18.0 (*ortho*-**C**H_3 of Mes) ppm.

Elemental Anal. found: C, 45.90; H, 5.43; N, 4.87 %; calculated for $C_{21}H_{28}ClIrN_2$ + H_2O: C, 45.52; H, 5.46; N, 5.06 %. ESI-MS (MeOH), m/z (%): 449.19 (100) $[M\text{-}Cl]^+$, 559.17 (55) $[M+Na]^+$ amu.

[Ir(MesIBn)CODCl] (**1d**)

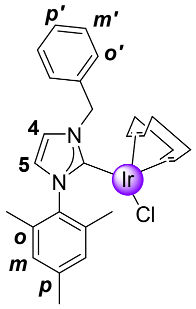

MesIBn.BPh_4 (101 mg, 0.170 mmol) was dissolved partially in acetone (20 mL). K_2CO_3 (127 mg, 0.920 mmol) was added and the suspension was stirred for 15 minutes at room temperature prior to the addition of $[Ir(COD)Cl]_2$ (57.2 mg, 0.0852 mmol). The bright orange mixture was heated at reflux for 4 hours, at which point the mixture looks orange-yellow in colour. After cooling to room temperature, the mixture was filtered through celite and evaporated to give an orange-yellow residue. The residue was redissolved in dichloromethane (20 mL), whereby white solid precipitates out. The mixture was filtered through celite and the solvent was evaporated to give an orange-yellow solid. The solid was then redissolved in hexane (30 mL) and heated with stirring in a water bath at about 40 °C for about 15 minutes. The yellow solution was filtered and collected. These two steps were repeated twice more until only a small amount of brown solid is remains undissolved. All the collected yellow solution were combined and reduced in volume to *ca.* 10 mL. The solution was cooled in the freezer overnight to give **1d** as yellow needle-like crystals.

Yield: 67.7 mg, 0.111 mmol, 65%

^1H NMR (CD_2Cl_2, 600 MHz, 298K): *δ* 7.41 (m, 4H, *ortho*- and *meta*-C**H** of phenyl ring), 7.35 (m, 1H, *para*-C**H** of phenyl ring), 7.04 (s, 1H, *meta*-C**H** of Mes), 6.97 (d, $^3J_{H\text{-}H}$ = 1.98 Hz, 1H, Im-**H**4), 6.94 (s, 1H, *meta*-C**H** of Mes), 6.80 (d, $^3J_{H\text{-}H}$ = 1.98 Hz, 1H, Im-**H**5), 6.10 (d, $^2J_{H\text{-}H}$ = 15.1 Hz, 1H, C**H**$_2$), 5.57 (d, $^2J_{H\text{-}H}$ = 15.1 Hz, 1H, C**H**$_2$), 4.37 (dt, $^3J_{H\text{-}H}$ = 11.8 Hz, $^3J_{H\text{-}H}$ = 3.94 Hz, 1H, =C**H** of COD), 4.30 (dt, $^3J_{H\text{-}H}$ = 11.8 Hz, $^3J_{H\text{-}H}$ = 3.94 Hz, 1H, =C**H** of

COD), 2.96 (dt, ${}^3J_{H\text{-}H}$ = 11.8 Hz, ${}^3J_{H\text{-}H}$ = 3.94 Hz, 1H, =C**H** of COD), 2.74 (dt, ${}^3J_{H\text{-}H}$ = 11.8 Hz, ${}^3J_{H\text{-}H}$ = 3.94 Hz, 1H, =C**H** of COD), 2.36 (s, 3H, *para*-C**H**$_3$ of Mes), 2.32 (s, 3H, *ortho*-C**H**$_3$ of Mes), 1.97 (m, 1H, C**H**$_2$ of COD), 1.92 (s, 3H, *ortho*-C**H**$_3$ of Mes), 1.89 (m, 1H, C**H**$_2$ of COD), 1.77 (m, 1H, C**H**$_2$ of COD), 1.51 (m, 1H, C**H**$_2$ of COD), 1.41 (m, 3H, C**H**$_2$ of COD), 1.18 (m, 1H, C**H**$_2$ of COD) ppm.

${}^{13}C\{{}^1H\}$ NMR (CD_2Cl_2, 150 MHz, 298K): δ 180.9 (Im-**C**2), 139.1 (*para*-**C**CH3 of Mes), 137.6 (**C**$_q$ of phenyl ring), 137.0 (*ortho*-**C**CH3 of Mes), 136.3 (**C**$_q$ of Mes), 134.9 (*ortho*-**C**CH3 of Mes), 129.6 (*meta*-**C**H of Mes), 129.1 (*ortho*-**C**H of phenyl ring), 128.5 (*meta*-**C**H of Mes overlapped with *meta*-**C**H of phenyl ring), 128.2 (*para*-**C**H of phenyl ring), 123.7 (Im-**C**5), 121.2 (Im-**C**4), 83.5 (=**C**H of COD), 83.1 (=**C**H of COD), 55.2 (**C**H$_2$), 52.6 (=**C**H of COD), 51.5 (=**C**H of COD), 34.5 (**C**H$_2$ of COD), 33.0 (**C**H$_2$ of COD), 29.7 (**C**H$_2$ of COD), 29.1 (**C**H$_2$ of COD), 21.2 (*para*-**C**H$_3$ of Mes), 19.6 (*ortho*-**C**H$_3$ of Mes), 18.0 (*ortho*-**C**H$_3$ of Mes) ppm.

HRMS (ESI$^+$, MeOH): *m/z* (%): (100 %) $[M\text{-}Cl]^+ = [C_{27}H_{32}IrN_2]^+$ = 577.2187 (Calculated $[M\text{-}Cl]^+$ = 577.2189) amu. Elemental Analysis: Found C, 52.75; H, 4.55, N, 4.43; Calculated for $C_{27}H_{32}ClIrN_2$: C, 52.97; H, 5.27; N, 4.58%.

[Ir(MesIEtPh)CODCl] (**1e**)

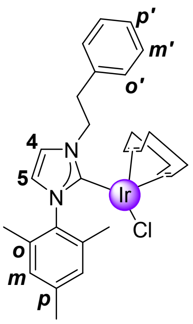

4.BPh_4 (107 mg, 0.176 mmol) was dissolved partially in acetone (20 mL). K_2CO_3 (127 mg, 0.0917 mmol) was added and the suspension was stirred for 15 minutes at room temperature prior to the addition of $[Ir(COD)Cl]_2$ (59.0 mg, 0.0878 mmol). The bright yellow mixture was heated at reflux for 4 hours, at which point the mixture looks orange-yellow in colour. After cooling to room temperature, the mixture was filtered through celite and evaporated to give an orange-yellow residue. The residue was redissolved in dichloromethane (20 mL), whereby white solid precipitates out. The mixture was filtered through celite and the solvent was evaporated to give an orange-yellow solid. The solid was then redissolved in hexane (30 mL) and heated with stirring in a water bath at about 40 °C for about 15 minutes. The yellow solution was filtered and collected. This two steps were repeated twice more until a small amount of brown solid is remains undissolved. All the collected yellow solution were

combined and reduced in volume to *ca.* 10 mL. The solution was cooled in the freezer overnight to give **1e** as yellow needle-like crystals.

Yield: 79.4 mg, 0.183 mmol, 72%

^{1}H NMR (CD_2Cl_2, 600 MHz, 298K): δ 7.28 (m, 5H, *ortho*-C**H**, *meta*-C**H** and *para*-C**H** of benzene ring), 7.03 (s, 1H, *meta*-C**H** of Mes), 6.94 (d, $^3J_{H-H}$ = 1.92 Hz, 1H, Im-**H**4), 6.92 (s, 1H, *meta*-C**H** of Mes), 6.74 (d, $^3J_{H-H}$ = 1.92 Hz, 1H, Im-**H**5), 5.13 (dt, $^2J_{H-H}$ = 13.5 Hz, $^3J_{H-H}$ = 7.68 Hz, 1H, ImN-C**H**$_2$), 4.46 (dt, $^2J_{H-H}$ = 13.5 Hz, $^3J_{H-H}$ = 7.68 Hz, 1H, ImN-C**H**$_2$), 4.38 (dt, $^3J_{H-H}$ = 11.6 Hz, $^3J_{H-H}$ = 3.56 Hz, 1H, =C**H** of COD), 4.29 (dt, $^3J_{H-H}$ = 11.6 Hz, $^3J_{H-H}$ = 3.56 Hz, 1H, =C**H** of COD), 3.30 (t, $^3J_{H-H}$ = 7.40 Hz, 2H, Ar-C**H**$_2$), 2.79 (dt, $^3J_{H-H}$ = 11.6 Hz, $^3J_{H-H}$ = 3.56 Hz, 1H, =C**H** of COD), 2.68 (dt, $^3J_{H-H}$ = 11.6 Hz, $^3J_{H-H}$ = 3.56 Hz, 1H, =C**H** of COD), 2.35 (s, 3H, *para*-C**H**$_3$), 2.28 (s, 3H, *ortho*-C**H**$_3$), 2.05 (m, 2H, C**H**$_2$ of COD), 1.87 (s, 3H, *ortho*-C**H**$_3$), 1.76 (m, 1H, C**H**$_2$ of COD), 1.48 (m, 3H, C**H**$_2$ of COD), 1.36 (m, 1H, C**H**$_2$ of COD), 1.18 (m, 1H, C**H**$_2$ of COD) ppm.

^{13}C{^{1}H} NMR (CD_2Cl_2, 150 MHz, 298K): δ 180.1 (Im-**C**2), 139.1 (*para*-**C**CH3 of Mes), 139.0 (**C**$_q$ of phenyl ring), 137.0 (*ortho*-**C**CH3 of Mes), 136.4 (**C**$_q$ of Mes), 135.0 (*ortho*-**C**CH3 of Mes), 129.6 (*meta*-**C**H of Mes), 129.4 (*meta*-**C**H of phenyl ring), 129.0 (*ortho*-**C**H of phenyl ring), 128.4 (*meta*-**C**H of Mes), 127.0 (*para*-**C**H of phenyl ring), 123.2 (Im-**C**5), 121.1 (Im-**C**4), 83.2 (=**C**H of COD), 82.7 (=**C**H of COD), 53.1 (ImN-**C**H$_2$), 52.2 (=**C**H of COD), 51.7 (=**C**H of COD), 37.5 (Ar-**C**H$_2$), 34.6 (**C**H$_2$ of COD), 33.1 (**C**H$_2$ of COD), 29.6 (**C**H$_2$ of COD), 29.3 (**C**H$_2$ of COD), 21.2 (*para*-**C**H$_3$ of Mes), 19.6 (*ortho*-**C**H$_3$ of Mes), 17.9 (*ortho*-**C**H$_3$ of Mes) ppm.

HRMS (ESI$^+$, MeOH): *m/z* (%): (100 %) $[M-Cl]^+$ = $[C_{28}H_{34}IrN_2]^+$ = 591.2347 (Calculated $[M-Cl]^+$ = 591.2346) amu. Elemental Analysis: Found C, 50.94; H, 5.55, N, 3.78; Calculated for $C_{28}H_{34}ClIrN_2.0.5CH_2Cl_2$: C, 51.19; H, 5.28; N, 4.19%.

Characterisation of active complexes in the presence of pyridine

[Ir(PhIMe)(py)$_3$(H)$_2$]Cl (**2b**)

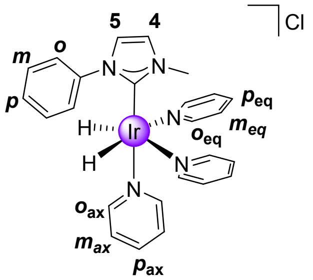

[Ir(PhIMe)(COD)Cl] (**1b**) (1.5 mg, 3.1 μmol) was dissolved in methanol-d_4 (0.6 mL) in a Young's tap NMR tube. Pyridine (1.3 μL, 0.016 mmol) was then added to the bright yellow mixture. The

mixture was degassed using three cycles of freeze-pump-thaw. *Para*hydrogen was added at a pressure of 3 bar and the sample was shaken to dissolve the hydrogen into solution. The bright yellow mixture turns very pale yellow in colour.

^{1}H NMR (CD_3OD, 400 MHz, 298K): δ 8.45 (d, $^{3}J_{H-H}$ = 6.23 Hz, 4H, *ortho*-C**H**, py *trans* to hydride), 7.85 (tt, J_{H-H} = 7.78 Hz, 1.77 Hz, 2H, *para*-C**H**, py *trans* to hydride), 7.66 (d, $^{3}J_{H-H}$ = 2.17 Hz, 1H, Im-**H**4/5), 7.36 (apparent t, J_{H-H} = 7.16 Hz, 4H, *meta*-C**H**, py *trans* to hydride), 7.15 (d, $^{3}J_{H-H}$ = 2.17 Hz, 1H, Im-**H**4/5), 7.10 (dd, J_{H-H} = 7.57 Hz, 1.18 Hz, 1H, *para*-C**H** of Ph), 6.88 (m, 2H, *ortho*-C**H** of Ph), 6.83 (q, 2H, *meta*-C**H** of Ph), 3.45 (s, 3H, C**H**$_3$), -22.14 (s, 2H, hydrides) ppm.

^{13}C{^{1}H} NMR (CD_3OD, 100 MHz, 298K): δ 153.8 (*ortho*-**C**H, py *trans* to hydride), 138.1 (*para*-**C**H, py *trans* to hydride), 127.1 (*meta*-**C**H, py *trans* to hydride), 125.4 (*meta*-**C**H of Ph), 123.22 (Im-**C**4/5), 122.8 (*ortho*-**C**H of Ph), 115.7 (Im-**C**4/5), 111.1 (*para*-**C**H of Ph), 27.5 (**C**H$_3$) ppm.

[Ir(MesIMe)(py)$_3$(H)$_2$]Cl (**2c**)

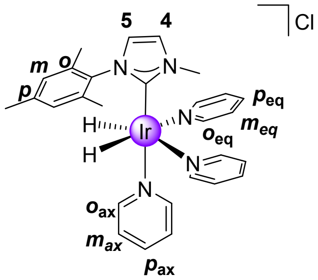

[Ir(MesIMe)(COD)Cl] (**1c**) (2.8 mg, 0.0031 mmol) was dissolved in methanol-d_4 (0.6 mL) in a Young's tap NMR tube. Pyridine (1.3 µL, 0.016 mmol) was then added to the bright yellow mixture. The mixture was degassed using three cycles of freeze-pump-thaw. *Para*hydrogen was added at a pressure of 3 bar and the sample was shaken to dissolve the hydrogen into solution. The bright yellow mixture turns colourless.

^{1}H NMR (CD_3OD, 400 MHz, 298K): δ 8.38 (d, $^{3}J_{H-H}$ = 4.69 Hz, 4H, *ortho*-C**H**, py *trans* to hydride), 8.26 (d, $^{3}J_{H-H}$ = 4.69 Hz, 2H, *ortho*-C**H**, py *cis* to hydride), 7.87 (tt, J_{H-H} = 8.48 Hz, 1.50 Hz, 2H, *para*-C**H**, py *trans* to hydride), 7.80 (tt, J_{H-H} = 7.64 Hz, 1.50 Hz, 1H, *para*-C**H**, py *cis* to hydride), 7.28 (d, $^{3}J_{H-H}$ = 2.05 Hz, 1H, Im-**H**4/5), 7.27 (m, 4H, *meta*-C**H**, py *trans* to hydride), 7.13 (m, 4H, *meta*-C**H**, py *cis* to hydride), 6.91 (d, $^{3}J_{H-H}$ = 2.05 Hz, 1H, Im-**H**4/5),

6.65 (s, 2H, *meta*-C**H** of Mes), 3.11 (s, 3H, C**H**$_3$), 2.21 (s, 3H, *para*-C**H**$_3$ of Mes), 1.99 (s, 6H, *ortho*-C**H**$_3$ of Mes), -22.34 (s, 2H, hydrides) ppm.

^{13}C{^{1}H} NMR (CD$_3$OD, 100 MHz, 298K): δ 155.5 (*ortho*-**C**H, py *cis* to hydride), 153.8 (*ortho*-**C**H, py *trans* to hydride), 138.3 (*para*-**C**CH$_3$ of Mes), 137.3 (*ortho*-**C**CH$_3$ of Mes), 136.5 (*para*-**C**H, py *cis* to hydride), 136.3 (*para*-**C**H, py *trans* to hydride), 135.7 (**C**$_q$ of Mes), 128.5 (2C, *meta*-**C**H of Mes), 125.9 (*meta*-**C**H of py *trans* to hydrides), 125.5 (*meta*-**C**H of py *cis* to hydrides), 122.5 (Im-**C**4/5), 122.02 (Im-**C**4/5), 36.9 (**C**H$_3$), 19.6 (*para*-**C**H$_3$ of Mes), 17.4 (2C, *ortho*-**C**H$_3$ of Mes) ppm.

[Ir(MesIBn)(py)$_3$(H)$_2$]Cl (**2d**)

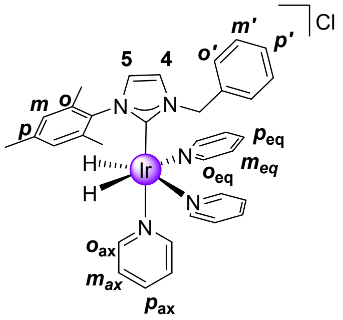

[Ir(MesIBn)(COD)Cl] (**1d**) (1.6 mg, 0.0026 mmol) was dissolved in methanol-d_4 (0.6 mL) in a Young's tap NMR tube. Pyridine (1.3 µL, 0.016 mmol) was then added to the bright yellow mixture. The mixture was degassed using three cycles of freeze-pump-thaw. *Para*hydrogen was added at a pressure of 3 bar and the sample was shaken to dissolve the hydrogen into solution. The bright yellow mixture turns colourless.

^{1}H NMR (CD$_3$OD, 400 MHz, 298K): δ 8.36 (d, $^3J_{H\text{-}H}$ = 4.59 Hz, 4H, *ortho*-C**H**, py *trans* to hydride), 8.22 (d, $^3J_{H\text{-}H}$ = 5.09 Hz, 2H, *ortho*-C**H**, py *cis* to hydride), 7.83 (tt, $J_{H\text{-}H}$ = 7.71 Hz, 1.48 Hz, 2H, *para*-C**H**, py *trans* to hydride), 7.78 (tt, $J_{H\text{-}H}$ = 7.69 Hz, 1.50 Hz, 1H, *para*-C**H**, py *cis* to hydride), 7.25 (apparent t, $J_{H\text{-}H}$ = 6.87 Hz, 3H, *meta*- and *para*-C**H** of Bn), 7.22 (m, 4H, *meta*-C**H**, py *trans* to hydride), 7.11 (m, 4H, *meta*-C**H**, py *cis* to hydride), 7.06 (d, $^3J_{H\text{-}H}$ = 1.93 Hz, 1H, Im-**H**4/5), 7.05 (d, $^3J_{H\text{-}H}$ = 7.74, 2H, *ortho*-C**H** of Bn), 6.95 (d, $^3J_{H\text{-}H}$ = 2.04 Hz, 1H, Im-**H**4/5), 6.67 (s, 2H, *meta*-C**H** of Mes), 4.89 (s, 2H, C**H**$_2$), 2.22 (s, 3H, *para*-C**H**$_3$ of Mes), 2.05 (s, 6H, *ortho*-C**H**$_3$ of Mes), -22.24 (s, 2H, hydrides) ppm.

^{13}C{^{1}H} NMR (CD$_3$OD, 100 MHz, 298K): δ 155.2 (*ortho*-**C**H, py *cis* to hydride), 153.8 (*ortho*-**C**H, py *trans* to hydride), and Cq of Bn find), 138.4 (*para*-**C**CH$_3$ of Mes), 136.9 (*ortho*-**C**CH$_3$ of Mes), 136.52 (*para*-**C**H, py *cis* to hydride), 136.34 (*para*-**C**H, py *trans* to hydride), 135.2 (**C**$_q$ of Mes) 128.5 (2C, *meta*-**C**H of Mes), *meta*- and *para*-**C**H of Bn), 126.6 (*ortho*-**C**H of Bn), 125.8 (*meta*-**C**H of py *trans* to hydrides) 125.5 (*meta*-**C**H of py *cis* to hydrides), 122.7 (Im-**C**4/5), 121.0 (Im-**C**4/5), 52.9 (**C**H$_2$), 19.5 (*para*-**C**H$_3$ of Mes), 17.4 (2C, *ortho*-**C**H$_3$ of Mes) ppm.

[Ir(MesIEtPh)(py)$_3$(H)$_2$]Cl (**2e**)

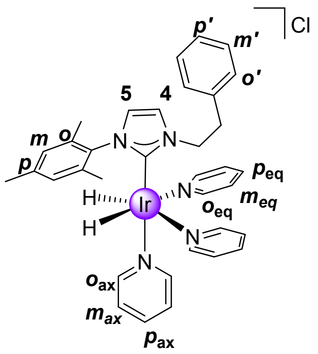

[Ir(MesIEtPh)(COD)Cl] (**1e**) (0.75 mg, 0.0012 mmol) was partially dissolved in methanol-d_4 (0.6 mL) in a Young's tap NMR tube. Pyridine (1.3 µL, 0.016 mmol) was then added to the yellow mixture. The mixture was heated in a water bath at 40 °C for 15 minutes to help dissolve the undissolved yellow solid. The mixture was degassed using three cycles of freeze-pump-thaw. *Para*hydrogen was added at a pressure of 3 bar and the sample was shaken to dissolve the hydrogen into solution. The bright yellow mixture turns colourless.

^{1}H NMR (CD$_3$OD, 400 MHz, 298K): δ 8.34 (d, $^3J_{H-H}$ = 5.03 Hz, 4H, *ortho*-C**H**, py *trans* to hydride), 8.25 (d, $^3J_{H-H}$ = 5.09 Hz, 2H, *ortho*-C**H**, py *cis* to hydride), 7.83 (tt, J_{H-H} = 7.74 Hz, 1.60 Hz, 2H, *para*-C**H**, py *trans* to hydride), 7.80 (tt, J_{H-H} = 7.62 Hz, 1.40 Hz, 1H, *para*-C**H**, py *cis* to hydride), 7.28 (d, $^3J_{H-H}$ = 1.83 Hz, 1H, Im-**H**4/5), 7.23 (m, 4H, *meta*-C**H**, py *trans* to hydride), 7.21 (m, 3H, *meta*- and *para*-C**H** of Bn), 7.13 (apparent t, J_{H-H} = 6.98 Hz, 4H, *meta*-C**H**, py *cis* to hydride), 7.03 (d, $^3J_{H-H}$ = 6.82, 2H, *ortho*-C**H** of Bn), 6.88 (d, $^3J_{H-H}$ = 1.83 Hz, 1H, Im-**H**4/5), 6.63 (s, 2H, *meta*-C**H** of Mes), 3.72 (t, $^3J_{H-H}$ = 7.92 Hz, 2H, Im-C**H**$_2$), 2.95 (t, $^3J_{H-H}$ = 7.99 Hz, 2H, Ph-C**H**$_2$), 2.20 (s, 3H, *para*-C**H**$_3$ of Mes), 1.99 (s, 6H, *ortho*-C**H**$_3$ of Mes), -22.31 (s, 2H, hydrides) ppm.

^{13}C{^{1}H} NMR (CD$_3$OD, 125 MHz, 298K): δ 155.6 (*ortho*-**C**H, py *cis* to hydride), 153.7 (*ortho*-**C**H, py *trans* to hydride), 138.4 (*para*-**C**CH$_3$ of Mes), 137.9 (Cq of Ph), 137.5 (*ortho*-**C**CH$_3$ of Mes), 136.9 (*para*-**C**H, py *cis* to hydride), 136.5 (*para*-**C**H, py *trans* to hydride), 135.1 (**C**$_q$ of Mes), 128.5 (2C, *meta*-**C**H of Mes), 128.3 (*ortho*-**C**H of Ph), 128.1 (*meta*-**C**H of Ph), 126.2 (*para*-**C**H of Ph), 125.8 (*meta*-**C**H of py *trans* to hydrides), 125.5 (*meta*-**C**H of py *cis* to hydrides), 122.2 (Im-**C**4/5), 120.8 (Im-**C**4/5), 51.3 (Im-**C**H$_2$), 35.6 (Ph-**C**H$_2$), 19.5 (*para*-**C**H$_3$ of Mes), 17.5 (2C, *ortho*-**C**H$_3$ of Mes) ppm.

Characterisation of the active complex formed in the presence of 3,4-lutidine

[Ir(PhIMe)(3,4-lutidine)$_3$(H)$_2$]Cl (**3b**)

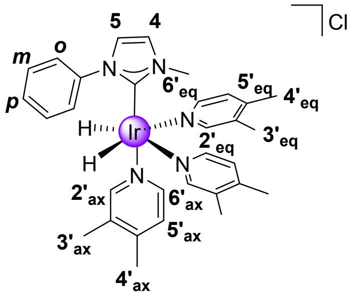

[Ir(PhIMe)(COD)Cl] (**1b**) (1.5 mg, 3.1 μmol) was dissolved in methanol-d_4 (0.6 mL) in a Young's tap NMR tube. 3,4-Lutidine (1.8 μL, 0.016 mmol) was then added to the bright yellow mixture. The mixture was degassed using three cycles of freeze-pump-thaw. *Para*hydrogen was added at a pressure of 3 bar and the sample was shaken to dissolve the hydrogen into solution. The bright yellow mixture turns colourless

^{1}H NMR (400 MHz, CD_3OD): *δ* 8.39 (s, 1H, **H**2' of 3,4-lutidine *cis* to hydride (axial position)), 8.13 (d, $^3J_{H\text{-}H}$ = 5.5 Hz, 2H, **H**6' of 3,4-lutidine *trans* to hydride (equatorial position)), 8.07 (s, 1H, **H**2' of 3,4-lutidine *trans* to hydride (equatorial position)), 7.65 (d, $^3J_{H4\text{-}H5}$ = 2.5 Hz, 1H, Im-**H**4), 7.18 (d, $^3J_{H\text{-}H}$ = 5.5 Hz, 1H, **H**6' of 3,4-lutidine *cis* to hydride (axial position)),7.15 (d, $^3J_{H4\text{-}H5}$ = 2.5 Hz, 1H, Im-**H**5), 7.13 (d, $^3J_{H\text{-}H}$ = 5.2 Hz, 2H, **H**5' of 3,4-lutidine *trans* to hydride (equatorial position)), 7.10 (d, $^3J_{H\text{-}H}$ = 5.6 Hz, 1H, **H**5' of 3,4-lutidine *cis* to hydride (axial position)), 6.87 (m, 3H, *o*- and *m*-C**H** of Ph), 6.81 (apparent t, $^3J_{H\text{-}H}$ = 7.0 Hz, 2H, *p*-C**H** of Ph), 3.46 (s, 3H, ImN-C**H**$_3$), -22.01 (s, 2H, hydrides) ppm.

[Ir(MesIMe)(3,4-lutidine)$_3$(H)$_2$]Cl (**3c**)

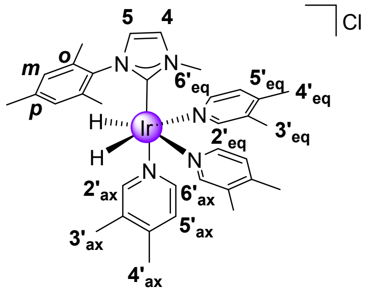

[Ir(MesIMe)(COD)Cl] (**1c**) (2.8 mg, 0.0052 mmol) was dissolved in methanol-d_4 (0.6 mL) in a Young's tap NMR tube. 3,4-Lutidine (1.7 μL, 0.015 mmol) was then added to the bright yellow mixture. The mixture was degassed using three cycles of freeze-pump-thaw. *Para*hydrogen was added at a pressure of 3 bar and the sample was shaken to dissolve the hydrogen into solution. The bright yellow mixture turns colourless.

^{1}H NMR (CD_3OD, 500 MHz, 298K): δ 8.12 (s, 1H, **H**2' of 3,4-lutidine *cis* to hydride), 8.04 (d, $^3J_{H\text{-}H}$ = 5.42 Hz, 2H, **H**6' of 3,4-lutidine *trans* to hydride), 8.00 (s, 1H, **H**2' of 3,4-lutidine *trans* to hydride), 7.72 (d, $^3J_{H\text{-}H}$ = 5.56 Hz, 1H, **H**6' of 3,4-lutidine *cis* to hydride), 7.25 (d, $^3J_{H\text{-}H}$ = 1.85 Hz, 1H, Im-**H**4/5), 7.05 (d, $^3J_{H\text{-}H}$ = 5.55 Hz, 2H, **H**5' of 3,4-lutidine *trans* to hydride), 6.87 (d, $^3J_{H\text{-}H}$ = 5.78 Hz, 1H, **H**5' of 3,4-lutidine *cis* to hydride), 6.83 (d, $^3J_{H\text{-}H}$ = 1.39 Hz, 1H, Im-**H**4/5), 6.65 (s, 2H, *meta*-C**H** of Mes), 3.13 (s, 3H, Im-C**H**$_3$), 2.28 (s, 6H,

CH_3-4’ of 3,4-lutidine *trans* to hydride), 2.21 (s, 3H, *para*-CH_3 of Mes), 2.19 (s, 3H, C**H**$_3$-4’ of 3,4-lutidine *cis* to hydride), 2.09 (s, 6H, C**H**$_3$-3’ of 3,4-lutidine *trans* to hydride), 2.07 (s, 3H, C**H**$_3$-3’ of 3,4-lutidine *cis* to hydride), 1.98 (s, 6H, *ortho*-CH_3 of Mes), -22.36 (s, 2H, hydrides) ppm.

[Ir(MesIBn)(3,4-lutidine)$_3$(H)$_2$]Cl (**3d**)

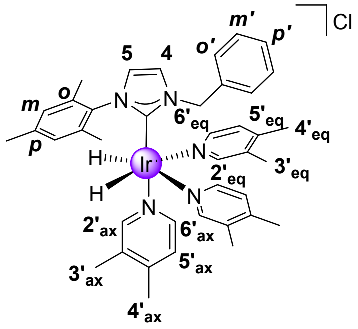

[Ir(MesIBn)(COD)Cl] (**1d**) (1.8 mg, 0.0031 mmol) was dissolved in methanol-d_4 (0.6 mL) in a Young’s tap NMR tube. 3,4-Lutidine (1.7 µL, 0.015 mmol) was then added to the bright yellow mixture. The mixture was degassed using three cycles of freeze-pump-thaw. *Para*hydrogen was added at a pressure of 3 bar and the sample was shaken to dissolve the hydrogen into solution. The bright yellow mixture turns colourless.

^{1}H NMR (CD_3OD, 400 MHz, 298K): δ 8.07 (s, 1H, **H**2’ of 3,4-lutidine *cis* to hydride), 8.05 (d, $^3J_{H-H}$ = 5.55 Hz, 2H, **H**6’ of 3,4-lutidine *trans* to hydride), 8.00 (s, 1H, **H**2’ of 3,4-lutidine *trans* to hydride), 7.23 (m, 3H, *meta*- and *para*-C**H** of Bn), 7.13 (d, $^3J_{H-H}$ = 1.62 Hz, 1H, Im-**H**4/5), 6.99 (apparent d, $^3J_{H-H}$ = 5.68 Hz, 4H, **H**5’ of 3,4-lutidine *trans* to hydride overlapped with , *ortho*-C**H** of Bn), 6.91 (d, $^3J_{H-H}$ = 1.62 Hz, 1H, Im-**H**4/5), 6.84 (d, $^3J_{H-H}$ = 5.68 Hz, 1H, **H**5’ of 3,4-lutidine *cis* to hydride), 6.70 (s, 2H, *meta*-C**H** of Mes), 4.97 (s, 2H, Im-C**H**$_2$), 2.24 (s, 6H, C**H**$_3$-4’ of 3,4-lutidine *trans* to hydride), 2.23 (s, 3H, *para*-CH_3 of Mes), 2.17 (s, 3H, C**H**$_3$-4’ of 3,4-lutidine *cis* to hydride), 2.06 (s, 6H, C**H**$_3$-3’ of 3,4-lutidine *trans* to hydride), 2.05 (s, 6H, *ortho*-CH_3 of Mes), 2.04 (s, 3H, C**H**$_3$-3’ of 3,4-lutidine *cis* to hydride), -22.30 (s, 2H, hydrides) ppm.

[Ir(MesIEtPh)(3,4-lutidine)$_3$(H)$_2$]Cl (**3e**)

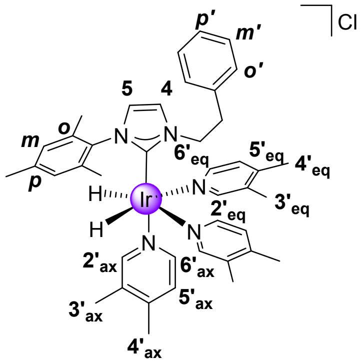

[Ir(MesIEtPh)(COD)Cl] (**1e**) (1.6 mg, 0.0026 mmol) was partially dissolved in methanol-d_4 (0.6 mL) in a Young’s tap NMR tube. 3,4-Lutidine (1.7 µL, 0.015 mmol) was then added to the yellow mixture. The mixture was heated in a water bath at 40 °C for 15 minutes to help dissolve the undissolved yellow

solid. The mixture was degassed using three cycles of freeze-pump-thaw. *Para*hydrogen was added at a pressure of 3 bar and the sample was shaken to dissolve the hydrogen into solution. The bright yellow mixture turns colourless.

^{1}H NMR (CD_3OD, 500 MHz, 298K): δ 8.12 (s, 1H, **H**2' of 3,4-lutidine *cis* to hydride), 8.05 (d, $^3J_{H-H}$ = 5.67 Hz, 2H, **H**6' of 3,4-lutidine *trans* to hydride), 8.00 (s, 1H, **H**2' of 3,4-lutidine *trans* to hydride), 7.71 (d, $^3J_{H-H}$ = 5.53 Hz, 1H, **H**6' of 3,4-lutidine *cis* to hydride), 7.26 (d, $^3J_{H-H}$ = 1.18 Hz, 1H, Im-**H**4/5), 7.20 (m, 3H, *meta*- and *para*-C**H** of Ph), 7.03 (d, $^3J_{H-H}$ = 5.45 Hz, 2H, **H**5' of 3,4-lutidine *trans* to hydride), 7.00 (d, $^3J_{H-H}$ = 7.35 Hz, 2H, *ortho*-C**H** of Bn), 6.87 (d, $^3J_{H-H}$ = 5.61 Hz, 1H, **H**5' of 3,4-lutidine *cis* to hydride), 6.83 (d, $^3J_{H-H}$ = 1.10 Hz, 1H, Im-**H**4/5), 6.65 (s, 2H, *meta*-C**H** of Mes), 3.75 (apparent t, $^3J_{H-H}$ = 8.07 Hz, 2H, Im-C$\mathbf{H}_2$), 2.92 (apparent t, $^3J_{H-H}$ = 8.07 Hz, 2H, Im-C$\mathbf{H}_2$), 2.25 (s, 6H, C$\mathbf{H}_3$-3' of 3,4-lutidine *trans* to hydride), 2.21 (s, 3H, *para*-CH_3 of Mes), 2.19 (s, 3H, C$\mathbf{H}_3$-3' of 3,4-lutidine *cis* to hydride), 2.07 (s, 3H, C$\mathbf{H}_3$-4' of 3,4-lutidine *cis* to hydride), 2.06 (s, 6H, C$\mathbf{H}_3$-4' of 3,4-lutidine *trans* to hydride), 1.99 (s, 6H, *ortho*-CH_3 of Mes), -22.36 (s, 2H, hydrides) ppm.

[Ir(IMes)(3,4-lutidine)$_3$(H)$_2$]Cl (**3a**)

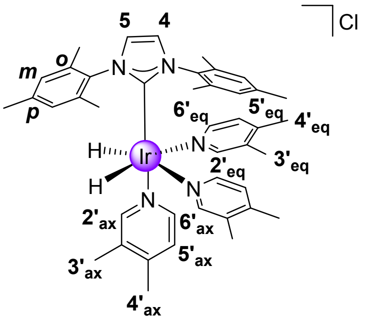

[Ir(IMes)(COD)Cl] (**1a**) (2.5 mg, 0.0026 mmol) was partially dissolved in methanol-*d*$_4$ (0.6 mL) in a Young's tap NMR tube. 3,4-Lutidine (1.7 μL, 0.015 mmol) was then added to the yellow mixture. The mixture was heated in a water bath at 40 °C for 15 minutes to help dissolve the undissolved yellow solid. The mixture was degassed using three cycles of freeze-pump-thaw. *Para*hydrogen was added at a pressure of 3 bar and the sample was shaken to dissolve the hydrogen into solution. The bright yellow mixture turns colourless.

^{1}H NMR (CD_3OD, 500 MHz, 298K): δ 8.06 (br s, 1H, **H**2' of 3,4-lutidine *cis* to hydride), 7.96 (br s, 2H, **H**6' of 3,4-lutidine *trans* to hydride), 7.91 (br s, 1H, **H**2' of 3,4-lutidine *trans* to hydride), 7.51 (d, $^3J_{H-H}$ = 4.14 Hz, 1H, **H**6' of 3,4-lutidine *cis* to hydride), 7.01 (s, 2H, Im-**H**4 and Im-**H**5), 6.91 (br s, 2H, **H**5' of 3,4-lutidine *trans* to hydride), 6.67 (br s, 5H, *meta*-C**H** of Mes overlapped with **H**5' of 3,4-lutidine *cis* to hydride), 2.25 (s, 3H, C$\mathbf{H}_3$-4' of 3,4-lutidine *trans* to hydride), 2.20 (s, 6H, *para*-CH_3 of Mes), 2.08 (s, 3H, C$\mathbf{H}_3$-4' of 3,4-lutidine

cis to hydride), 2.05 (s, 3H, C**H**$_3$-3’ of 3,4-lutidine *trans* to hydride), 2.03 (s, 12H, *ortho*-CH$_3$ of Mes), 1.96 (s, 3H, C**H**$_3$-3’ of 3,4-lutidine *cis* to hydride), -22.82 (s, 2H, hydrides) ppm.

Characterisation of the active complex formed in the presence of 3,5-lutidine

[Ir(PhIMe)(3,5-lutidine)$_3$(H)$_2$]Cl (**4b**)

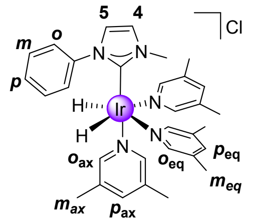

Catalytic reaction quantities: [Ir(PhIMe)(COD)Cl] (**1b**) (1.3 mg, 2.6 μmol) and 3,5-lutidine (1.7 μL, 0.015 mmol) in methanol-d_4 (0.6 mL).

^{1}H NMR (CD$_3$OD, 400MHz, 298K): δ 8.03 (s, 4H, *o*-C**H** of 3,5-lutidine *trans* to hydrides (equatorial position)), 7.66 (d, $^3J_{H4\text{-}H5}$ = 2.0 Hz, 1H, Im-**H**4), 7.64 ((s, 2H, *p*-C**H** of 3,5-lutidine *cis* to hydrides (axial position)), 7.54 (s, 2H, *p*-C**H** *trans* to 3,5-lutidine (equatorial position)), 7.16 (d, $^3J_{H4\text{-}H5}$=2.0 Hz, 1H, Im-**H**5), 7.10 (d, $^3J_{H\text{-}H}$=7.35 Hz, 1H *p*-C**H** of Ph), 6.83 (m, 4H, *o*-and *m*-C**H** of Ph), 6.82 (s, H, *p*-C**H** of 3,5-lutidine *cis* to hydrides (axial position)), 3.46 (s, 3H,-NC**H**$_3$), 2.29 (s, 12H, *m*-C**H**$_3$ of 3,5-lutidine *trans* to hydrides (equatorial position)), 2.20 (s, 6H, *m*-C**H**$_3$ of 3,5-lutidine *cis* to hydrides (axial position)), -22,24 (s, 2H, hydrides) ppm. **^{13}C{^{1}H} NMR** (500 MHz, CD$_3$OD, 303 K): δ 154.9 (Im-**C**2), 149.3 (*o*-CH of 3,5-lutidine *trans* to hydrides (equatorial position)), 148.4 (*ipso*-**C**$_q$ of Ph), 148.3 (*p*-CH of 3,5-lutidine *cis* to hydrides (axial position)), 138.6 (*o*- and *m*-CH of Ph), 137.9 (*p*-**C**H of 3,5-lutidine *trans* to hydrides (equatorial position)), 135.9 (*m*-**C**CH$_3$ of 3,5-lutidine *cis* to hydrides (axial position)), 135.2 (*m*-**C**CH$_3$ of 3,5-lutidine *trans* to hydrides (equatorial position)), 133.0 (*o*-**C**H of 3,5-lutidine *cis* to hydrides (axial position)), 121.8 (Im-**C**5), 121.3 (Im-**C**4), 109.8 (*p*-**C**H of Ph), 34.6 (ImN-**C**H$_3$), 18.01 (*m*-**C**H$_3$ of 3,5-lutidine *trans* to hydrides (equatorial position)),16.52 (*m*-**C**H$_3$ of 3,5-lutidine *cis* to hydrides (axial position)) ppm.

[Ir(MesImMe)(3,5-lutidine)$_3$(H)$_2$]Cl (**4c**)

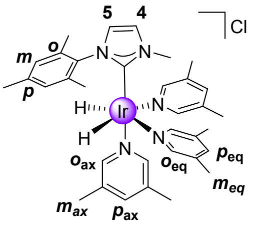

Catalytic reaction quantities: [Ir(MesIMe)(COD)Cl] (**1c**) (1.6 mg, 2.6 μmol) and 3,5-lutidine (1.7 μL, 0.015 mmol) in methanol-d_4 (0.6 mL).

^{1}H NMR (CD_3OD, 400 MHz, 298K): δ 7.99 (s, 4H, *o*-C**H** of 3,5-lutidine *trans* to hydrides (equatorial position)), 7.88 (s, 2H, *o*-C**H** of 3,5-lutidine *cis* to hydrides (axial position)), 7.56 (s, 2H, *p*-C**H** of 3,5-lutidine *trans* to hydrides (equatorial position)), 7.49 (s, 1H, *p*-C**H** of 3,5-lutidine *cis* to hydrides (axial position)), 7.30 (d, $^{3}J_{H\text{-}H}$=2.0 Hz ,1H, Im-**H**4), 6.88 (d, $^{3}J_{H\text{-}H}$=2.0 Hz ,1H, Im-**H**5), 6.69 (s, 2H, *m*-C**H** of Mes), 3.18 (s, 3H, ImN-C**H**$_3$), 2.23 (s, 6H, *o*-C**H**$_3$ of Mes), 2.20 (s, 12H, *m*-C**H**$_3$ of 3,5-lutidine *trans* to hydrides (equatorial position)), 2.11 (s, 3H, *p*-C**H**$_3$ of Mes), 2.01 (s, 6H, *m*-C**H**$_3$ of 3,5-lutidine *cis* to hydrides (axial position)), -22.35 (s, 2H, hydrides) ppm. **^{13}C{^{1}H} NMR** (500 MHz, CD_3OD, 303 K): δ 158.9 (Im-**C**2), 152.7 (*p*-**C**H of 3,5-lutidine *cis* to hydrides (axial position)), 151.2 (*p*-**C**H of 3,5-lutidine *trans* to hydrides (equatorial position)), 138.8 (*o*-**C**H of 3,5-lutidine *trans* to hydrides (equatorial position)), 138.6 (*p*-**C**CH$_3$ of Mes), 137.9 (*o*-CH of 3,5-lutidine *cis* to hydrides (axial position)), 137.8 (*o*-**C**CH of Mes), 136.5 (*ipso*-**C**$_q$ of Mes), 135.9 (*m*-**C**CH$_3$ of 3,5-lutidine *cis* to hydrides (axial position)), 135.2 (*m*-**C**CH$_3$ of 3,5-lutidine *trans* to hydrides (equatorial position)), 128.5 (*m*-**C**H of Mes), 128.4 (Im-**C**4), 122.0 (Im-**C**5), 37.8 (ImN-**C**H$_3$), 37.0 (*p*-**C**H$_3$ of Mes), 19.7 (*o*-**C**H$_3$ of Mes), 17.2 (*m*-**C**H$_3$ of 3,5-lutidine *trans* to hydrides (equatorial position)), 16.7 (*m*-**C**H$_3$ of 3,5-lutidine *cis* to hydrides (axial position)) ppm.

[Ir(MesIBn)(3,5-lutidine)$_3$(H)$_2$]Cl (**4d**)

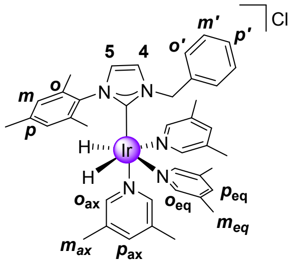

Catalytic reaction quantities: [Ir(MesIBn)(COD)Cl] (**1d**) (1.6 mg, 2.6 µmol) and 3,5-lutidine (1.7 µL, 0.015 mmol) in methanol-d_4 (0.6 mL).

^{1}H NMR (CD_3OD, 400 MHz, 298K): δ 7.99 (s, 4H, *o*-C**H** of 3,5-lutidine *trans* to hydrides (equatorial position)), 7.82 (s, 2H, *o*-C**H** of 3,5-lutidine *cis* to hydrides (axial position)), 7.48 (s, 2H, *p*-C**H** of 3,5-lutidine *trans* to hydrides (equatorial position)), 7.46 (s, 1H, *p*-C**H** of 3,5-lutidine *cis* to hydrides (axial position)), 7.27 (dt, $^{3}J_{H\text{-}H}$=8.3 Hz, $^{3}J_{H\text{-}H}$=7.2 Hz 3H, *p*-C**H** of phenyl ring overlapped with *m*-C**H** of phenyl ring), 7.04 (d, $^{3}J_{H\text{-}H}$=5.49 Hz, 2H, *o*-C**H** of phenyl ring), 7.17 (s, 1H, Im-**H**4), 6.94 (s, 1H, Im-**H**5), 6.73 (s, 2H, *m*-C**H** of Mes), 5.01 (s, 2H, ImN-C**H**$_2$-), 2.25 (s, 3H, *p*-C**H**$_3$ of Mes), 2.16 (s, 6H, *m*-C**H**$_3$ of 3,5-lutidine *cis* to hydrides (axial position)), 2.14 (s, 6H, *o*-C**H**$_3$ of Mes), 2.07 (s, 12H, *m*-C**H**$_3$ of 3,5-lutidine *trans* to hydrides (equatorial position)), -22.28 (s, 2H, hydrides) ppm. **^{13}C{^{1}H} NMR** (400 MHz, CD_3OD): δ 159.9 (Im-**C**2), 152.5 (*p*-**C**H of 3,5-lutidine *cis* to hydrides (axial position)), 150.9 (*p*-**C**H of 3,5-lutidine *trans* to hydrides (equatorial

position)), 145.9 (*ipso*-$\mathbf{C}_q$ of phenyl ring), 138.7 (*p*-**C**CH_3 of Mes), 138.5 (*o*-**C**H of 3,5-lutidine *trans* to hydrides (equatorial position)), 137.7 (*o*-**C**CH_3 of Mes), 135.7 (*m*-**C**CH_3 of 3,5-lutidine *cis* to hydrides (axial position)), 135.4 (*ipso*-$\mathbf{C}_q$ of Mes), 135.0 (*m*-**C**CH_3 of 3,5-lutidine *trans* to hydrides (equatorial position)), 134.7 (*o*-**C**H of 3,5-lutidine *cis* to hydrides (axial position)), 128.3 (*m*-**C**H of Mes), 127.9 (*m*-**C**H of phenyl ring), 126.9 (*p*-**C**H of phenyl ring), 126.3 (*o*-**C**H of phenyl ring), 122.7 (Im-**C**4), 121.2 (Im-**C**5), 52.8 (ImN-**C**H_2-), 26.3 (*p*-**C**H_3 of Mes), 19.9 (*o*-**C**H_3 of Mes), 16.7 (*m*-**C**H_3 of 3,5-lutidine *trans* to hydrides (equatorial position)), 16.5 (*m*-**C**H_3 of 3,5-lutidine *cis* to hydrides (axial position)) ppm.

[Ir(MesIEtPh)(3,5-lutidine)$_3$(H)$_2$]Cl (**4e**)

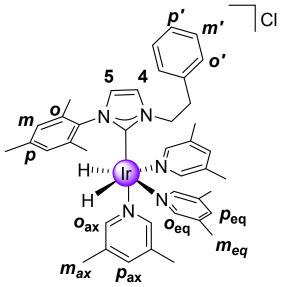

Catalytic reaction quantities: [Ir(MesIEtPh)(COD)Cl] (**1e**) (1.6 mg, 2.6 μmol) and 3,5-lutidine (1.7 μL, 0.015 mmol) in methanol-d_4 (0.6 mL). The mixture was heated in a water bath at 40 °C for 15 minutes to help dissolve the undissolved yellow solid before *p*-H_2 was added.

^{1}H NMR (CD_3OD, 500 MHz, 298K): *δ* 8.00 (s, 4H, *o*-C**H** of 3,5-lutidine *trans* to hydrides (equatorial position)), 7.88 (s, 2H, *o*-C**H** lutidine in axial position), 7.51 (s, 2H, *p*-C**H** of 3,5-lutidine *trans* to hydrides (equatorial position)), 7.47 (s, 1H, *p*-C**H** of 3,5-lutidine *cis* to hydrides (axial position)), 7.31 (d, $^3J_{H\text{-}H}$= 2.0 Hz, 1H, Im-**H**4), 7.24 (d, $^3J_{H\text{-}H}$ = 7.2 Hz, 2H, *m*-C**H** of phenyl ring), 7.19 (d, $^3J_{H\text{-}H}$=7.3 Hz, 1H, *p*-C**H** of phenyl ring), 7.03 (d, $^3J_{H\text{-}H}$ = 7.4 Hz, 2H, *o*-C**H** of phenyl ring), 6.88 (d, $^3J_{H\text{-}H}$= 2.0 Hz, 1H, Im-**H**5), 6.69 (s, 2H, *m*-C**H** of Mes), 3.76 (t, $^3J_{H\text{-}H}$= 7.8 Hz, 2H, ImN-C**H**$_2$-), 2.96 (t, $^3J_{H\text{-}H}$= 7.8 Hz, 2H, ImN-CH_2C**H**$_2$-), 2.23 (s, 3H, *p*-C**H**$_3$ of Mes), 2.17 (s, 12H, *m*-C**H**$_3$ of 3,5-lutidine *trans* to hydride (equatorial position)), 2.11 (s, 6H, *m*-C**H**$_3$ of 3,5-lutidine *cis* to hydrides (axial position)), 2.02 (s, 6H, *o*-C**H**$_3$ of Mes), -22.38 (s, 2H, hydrides) ppm. **^{13}C{^{1}H} NMR** (500 MHz, CD_3OD): *δ* 158.9 (Im-**C**2), 152.8 (*p*-**C**H of 3,5-lutidine *cis* to hydrides (axial position)), 151.1 (*p*-**C**H of 3,5-lutidine *trans* to hydride (equatorial position)), 138.2 (*p*-**C**CH_3 of Mes), 138.1 (*ipso*-$\mathbf{C}_q$ of phenyl ring), 137.8 (*o*-**C**H of 3,5-lutidine *trans* to hydride (equatorial position)), 137.6 (*o*-**C**CH_3 of Mes), 135.9 (*m*-**C**CH_3 of 3,5-lutidine *cis* to hydrides (axial position)), 135.3 (*ipso*-$\mathbf{C}_q$ of Mes), 135.2 (*m*-**C**CH_3 of 3,5-lutidine *trans* to hydride (equatorial position)), 134.9 (*o*-**C**H of 3,5-lutidine *cis* to hydrides (axial position)), 128. 5 (*m*-**C**H of Mes), 128.3 (*o*-**C**H of phenyl ring), 128.1 (*m*-**C**H of phenyl ring), 126.0 (*p*-**C**H of phenyl ring), 122.2 (Im-**C**4), 120.5 (Im-**C**5), 51.8 (ImN-**C**H_2-), 35.1 (ImN-CH_2**C**H_2-), 19.9

(*p*-CH$_3$ of Mes), 17.3 (*o*-**C**H$_3$ of Mes), 16.7 (*m*-CH$_3$ of 3,5-lutidine *trans* to hydride (equatorial position)), 16.5 (*m*-**C**H$_3$ of 3,5-lutidine *cis* to hydrides (axial position)) ppm.

<u>[Ir(IMes)(3,5-lutidine)$_3$(H)$_2$]Cl (**4a**)</u>

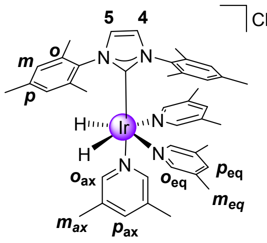

Catalytic reaction quantities: [Ir(IMes)(COD)Cl] (**1a**) (1.7 mg, 2.6 µmol) and 3,5-lutidine (1.7 µL, 0.015 mmol) in methanol-d_4 (0.6 mL).

^{1}H NMR (CD$_3$OD, 500 MHz, 298K): δ 7.99 (s, 4H, *o*-C**H** of 3,5-lutidine *trans* to hydride (equatorial position)), 7.88 (s, 2H, *o*-C**H** of 3,5-lutidine *cis* to hydride (axial position)), 7.55 (s, 2H, *p*-C**H** of 3,5-lutidine *trans* to hydride (equatorial position)), 7.49 (s, H, *p*-C**H** of 3,5-lutidine *cis* to hydride (axial position)), 7.30 (s, 2H, Im-**H**4 and Im-**H**5), 6.93 (s,4H, *m*-C**H** of Mes), 2.23 (s, 6H, *p*-C**H**$_3$ of Mes), 2.19 (s, 12H, *m*-C**H**$_3$ of 3,5-lutidine *trans* to hydride (equatorial position)), 2.10 (s, 12H, *o*-C**H**$_3$ of Mes), 2.01 (s, 6H, *m*-CH$_3$ of 3,5-lutidine *cis* to hydride (axial position)), -22.79 (s, 2H, hydrides). **^{13}C{^{1}H} NMR** (500 MHz, CD$_3$OD): δ 152.4 (*p*-**C**H of 3,5-lutidine *cis* to hydride (axial position)), 152.2 (Im-**C**2), 152.3 (*p*-**C**H of 3,5-lutidine *trans* to hydride (equatorial position)), 137.5 (*o*-**C**H of 3,5-lutidine *trans* to hydride (equatorial position)), 135.9 (*m*-**C**CH$_3$ of 3,5-lutidine *cis* to hydride (axial position)), 135.2 (*m*-**C**CH$_3$ of 3,5-lutidine *trans* to hydride (equatorial position)), 134.9 (*o*-**C**H of 3,5-lutidine *cis* to hydride (axial position)), 137.9 (*p*-**C**CH$_3$ of Mes), 137.7 (*o*-**C**CH$_3$ of Mes), 135.3 (*ipso*-**C**$_q$ of Mes), 128.2 (*m*-**C**H of Mes), 122.4 (Im-**C**4 and Im-**C**5), 19.7 (*p*-**C**H$_3$ of Mes), 17.4 (*o*-**C**H$_3$ of Mes), 16.7 (*m*-**C**H$_3$ of 3,5-lutidine *trans* to hydride (equatorial position)), 16.1 (*m*-**C**H$_3$ of 3,5-lutidine *cis* to hydride (axial position)).

<u>Ligand exchange rates and proton NMR signal enhancement</u>

Table 1S: Full list of ligand exchange rates and errors at the indicated temperature.

**complex**	**pyridine**			**hydride**			**3,4-lutidine**		
	298 K	303 K	313 K	298 K	303 K	313 K	298 K	303 K	313 K
**1a**	13.3±0.4	28.5±0.9	88±5				9.72±0.04	17.10±0.04	-

**1b**	-	-	-	-	-	-	-	-	-
**1c**	0.508±0.005	0.884±0.002	3.662±0.002	0.183±0.004	-	1.180±0.010	0.390±0.002	0.785±0.004	2.70±0.02
**1d**	0.713±0.003	1.70±0.01	6.768±0.010	0.0384±0.007	-	-	0.764±0.008	1.465±0.002	4.66±0.09
**1e**	1.23±0.03	1.940±0.006	7.26±0.09	0.780±0.01	0.87±0.02	1.43±0.04	0.714±0.004	1.56±0.05	5.02±0.05

***288 K k_{diss}=3.55 ± 0.03 s^{-1}, total enhancement = 3200. It should be note that the k_{diss} rates correspond to the rate constant for the loss of one of the two axial ligands.

Table 2S: Full list of attributed signals enhancements for the *ortho*-, *meta*- and *para*- protons of pyridine and the total signal enhancement.

	**pyridine at 298K**				**pyridine at 313K**			
	ortho-H	meta-H	para-H	total enhancement	ortho-H	meta-H	para-H	total enhancement
**1b**	-0.77	-0.14	-0.32	2.32				
**1c**	-48.2	-11.8	-27.2	162.6	-158.1	-70.2	-87.4	561.2
**1d**	-95.2	-32.8	-47.8	318.8	-196.4	-82.6	-119.2	713.8
**1e**	-68.8	-15	-40.2	233.0	-129.6	-57	-76.5	469.2

Table 3S. Full list of attributed signal enhancements observed for 3,4-lutidine, and the total signal enhancement.

	**3,4-lutidine at 298K**	**3,4-lutidine at 313K**

	H2 and H6	H5	CH_3 3 and 4	total	H2 and H6	H5	CH_3 3 and 4	total
**1a**	-540	-184	-223	1709	-1150	-715	-607	3080
**1b**								
**1c**	-138	-54	-70	471	-167	-59	-89	570
**1d**	-173	-56	-79	506	-212	-76	-108	716
**1e**	-67	-24	-33	223	-170	-62	-85	572
**1**	-540	-184	-223	1709	-1150	-715	-607	3080
**1 at 288k**	-1800	679	-728	3207				

Table 4S. Full list of enhancement of 3,5-lutidine and the total enhancement

	**3,5-lutidine at 298K**			
	H2 and H6	H4	CH_3 3 and 5	total
**1a**	-290	27	66	740
**1b**	-3.	-1	-1	11
**1c**	-250	-75	-71	719
**1d**	-516	-230	-176	1615
**1e**	-173	-91	-67	504

SABRE experiments, using Flow-apparatus

^{1}H NMR enhancements in the flow-apparatus

- Pyridine

Table 5S. Signal enhancement levels for the *ortho*, *para* and *meta* protons respectively of pyridine observed when using catalyst **1d**.

Field	Signal Enhancement (Fold)		
	ortho	*para*	*meta*
0	-19	-14	25
10	-72	-51	41
20	-73	-46	14
30	-73	-45	-3

40	-93	-57	-14
50	-130	-80	-49
60	-150	-96	-78
70	-175	-107	-95
80	-165	-100	-82
90	-117	-74	-48
100	-97	-63	-44
110	-81	-55	-60
120	-85	-54	-68
130	-79	-50	-39
140	-69	-46	-9

- 3,4-lutidine

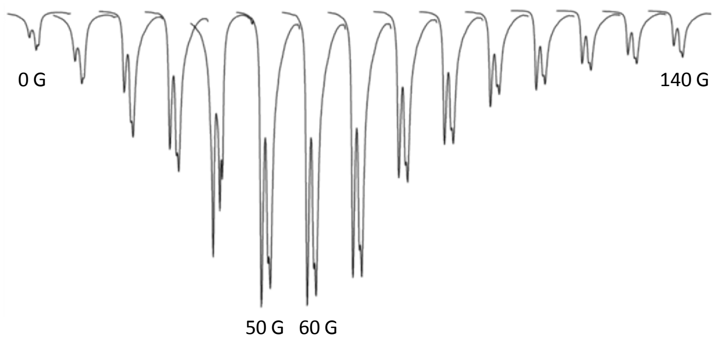

Figure 1S. Intensity of the H2 and H6 proton responses of 3,4-lutidine as a function of polarisation transfer field over the range 0 G to 140 G, in steps of 10 G.

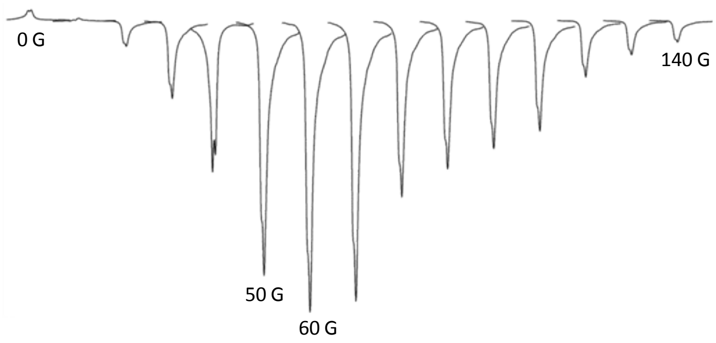

Figure 2S. Intensity of the H5 proton response of 3,4-lutidine as a function of polarisation transfer field over the range 0 G to 140 G, in steps of 10 G.

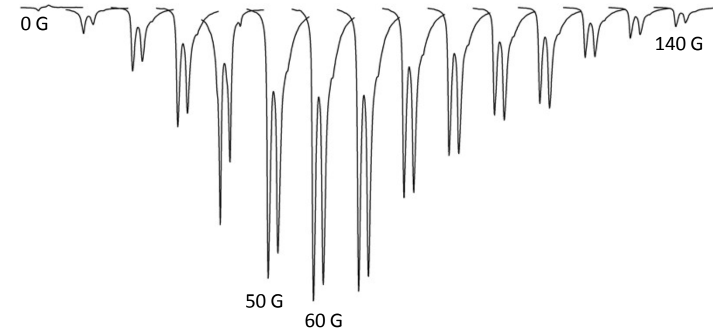

Figure 3S. Intensity of CH_3 protons at position 3 and 4 of 3,4-lutidine as a function of polarisation transfer field over the range 0 G to 140 G, in steps of 10 G.

.

Table 6S. ^{1}H NMR signal enhancements for H2, H5, H6, 3-CH_3 and 4-CH_3 respectively of 3,4-lutidine observed when using catalyst **1d** in the flow apparatus.

Field (G)	Enhancement		
	**H2 and H6**	**H5**	**CH3 3 and 4**
0	-33	4	-0.1
10	-64	0.1	-13
20	-89	-7	-22
30	-124	-20	-42
40	-178	-44	-87
50	-231	-65	-97
60	-233	-74	-105

70	-216	-72	-103
80	-138	-45	-70
90	-109	-38	-54
100	-70	-31	-39
110	-60	-27	-34
120	-45	-14	-18
130	-40	-9	-11
140	-34	-6	-6

-3,5-lutidine

Table 7S. ^{1}H NMR signal enhancements for H2, H5, H6, 3-CH_3 and 4-CH_3 respectively of 3,5-lutidine observed when using catalyst **1d** in the flow apparatus.

Field (G)	Enhancement		
	**H2 and H6**	**H4**	**CH3 3 and 5**
0	-41	-7	18
10	-91	-20	-3
20	-151	-40	-36
30	-280	-73	-109
40	-419	-125	-188

50	-574	-210	-312
60	-671	-242	-408
70	-695	-302	-490
80	-691	-339	-508
90	-459	-222	-343
100	-348	-162	-252
110	-254	-110	-161
120	-218	-89	-110
130	-184	-67	-68
140	-160	-53	-40

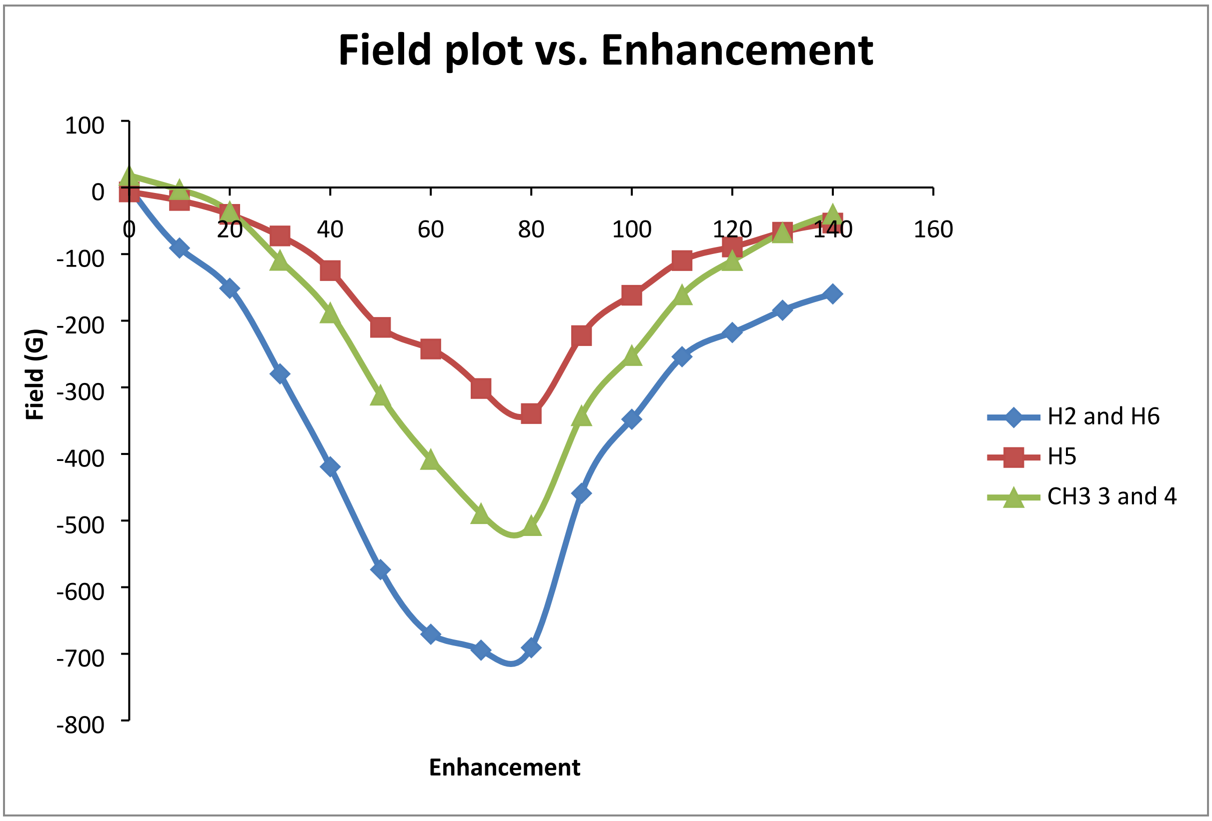

Figure 4S. Intensity of the indicated protons of 3,5-lutidine as a function of polarisation transfer field over the range 0 G to 140 G, in steps of 10 G.

^{13}C NMR enhancements data

- 3,4-lutidine

Complex **1a**

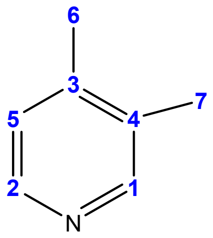

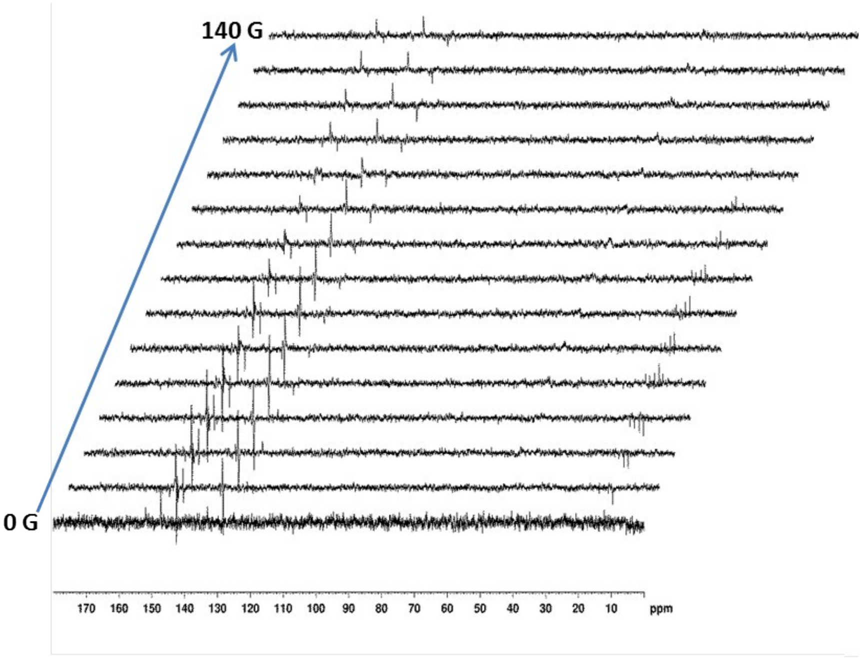

Complex **1d**

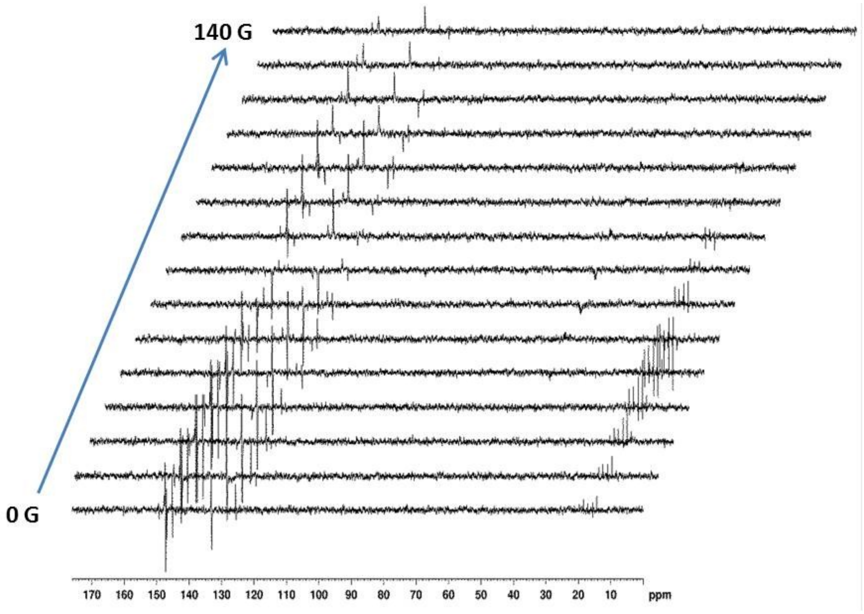

Figure 5S. Stack plot showing how the intensity of the polarised carbon peaks observed for 3,4-lutidine vary when using complex **1a** and **1d** respectively. Spectra were recorded in steps of 10 G over a polarisation field transfer field range of 0 G to 140 G.

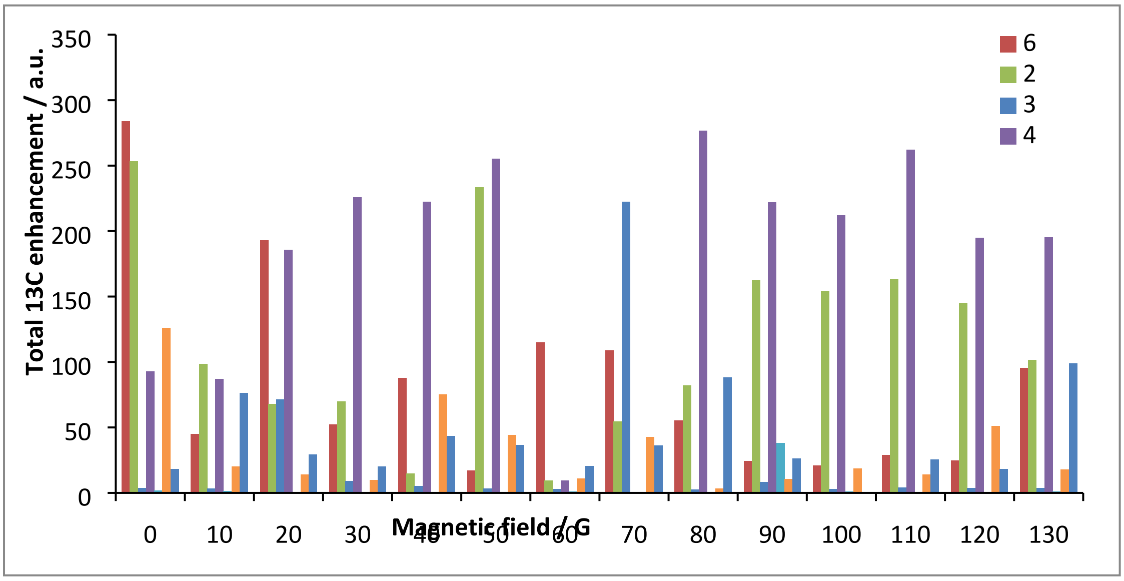

Figure 6S. Polarization transfer field profile for the indicated ^{13}C NMR signals of 3,4-lutidine achieved when using complex **1d**.

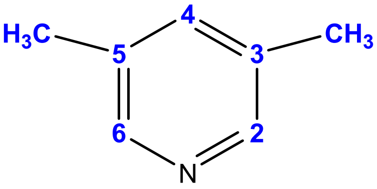

3,5-lutidine

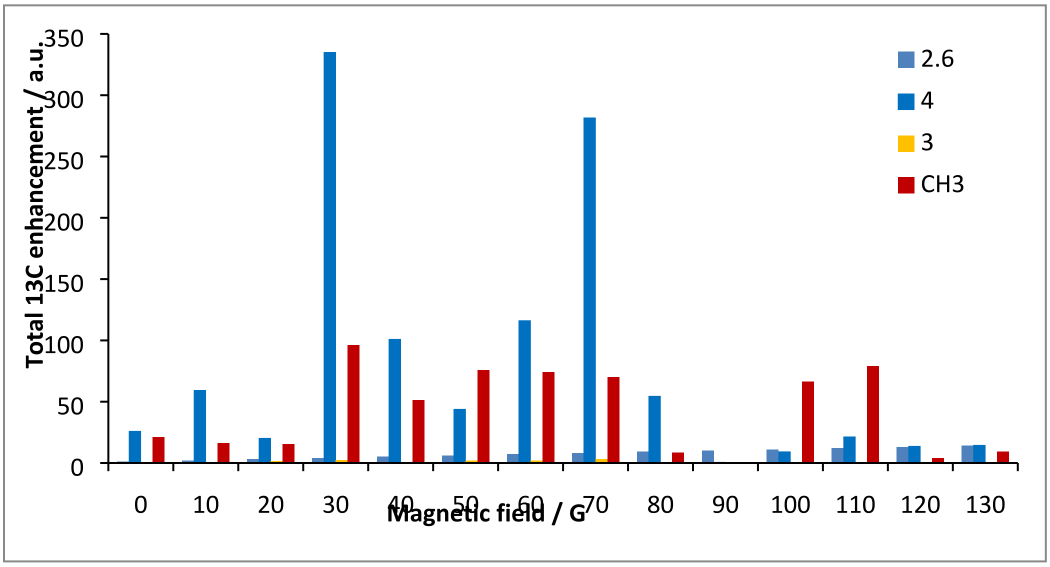

Figure 7S. Polarization transfer field profile for the polarised ^{13}C NMR signals of 3,5-lutidine achieve with complex **1d**.

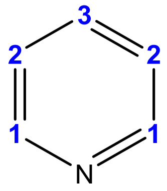

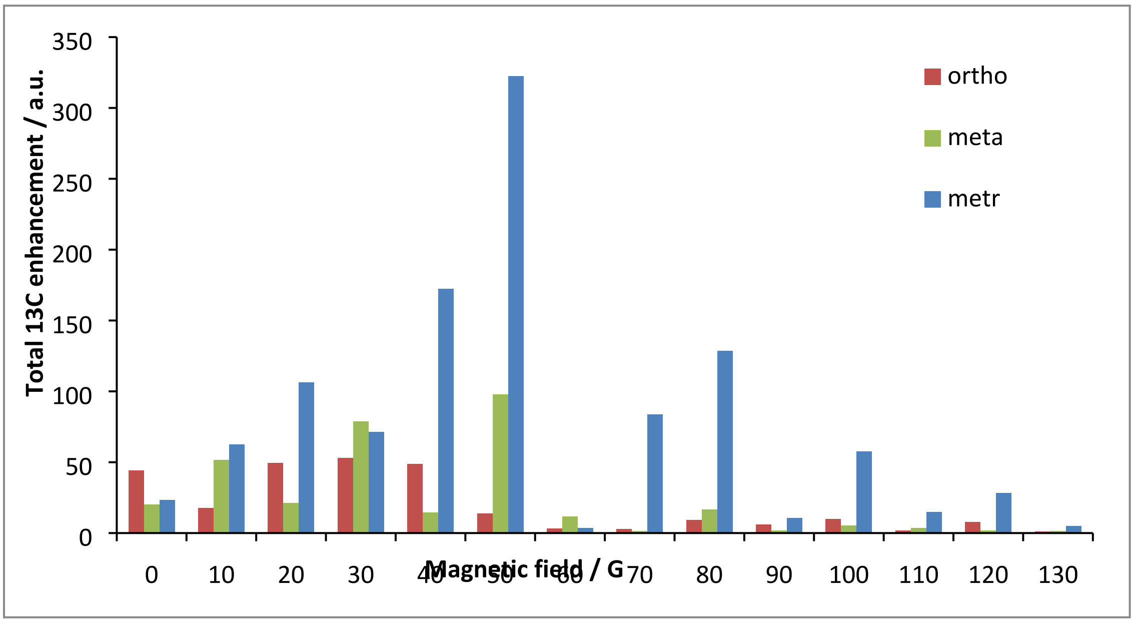

Figure 8S. Polarization transfer field profile of the polarised ^{13}C NMR signals of pyridine achieved with complex **1a**.

Reactions of **1** with pyridine.

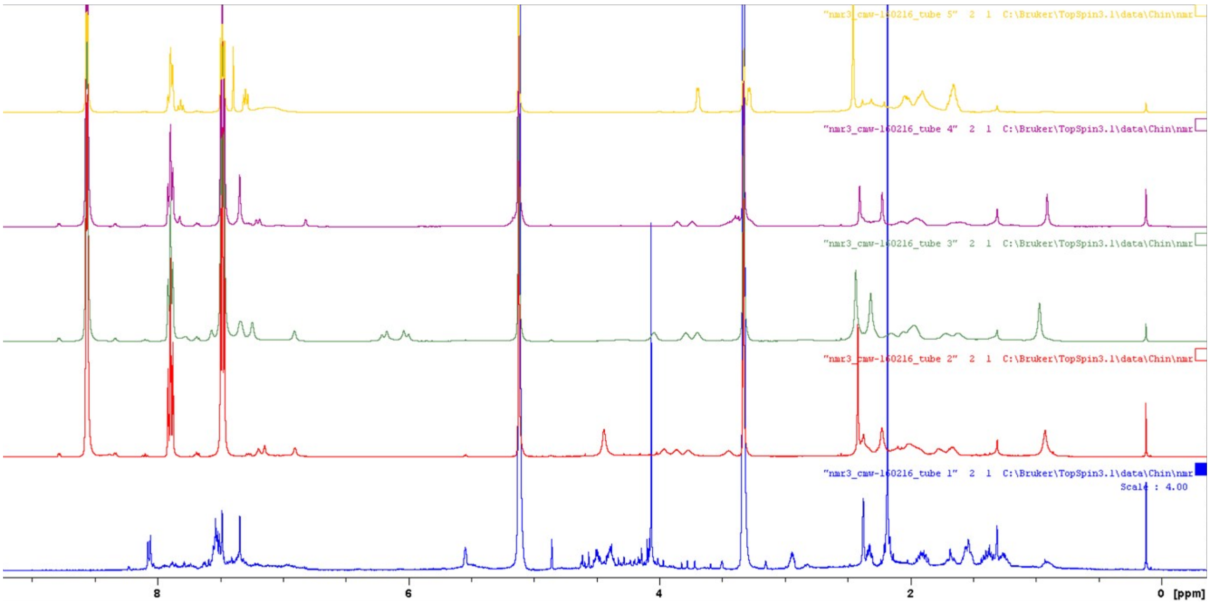

Figure 9S. NMR spectra detailing the changes associated with the addition of pyridine to samples of **1**. Conversion to [Ir(py)(NHC)(COD)]Cl is only evident for **1a**.

X-Ray analysis of 1e

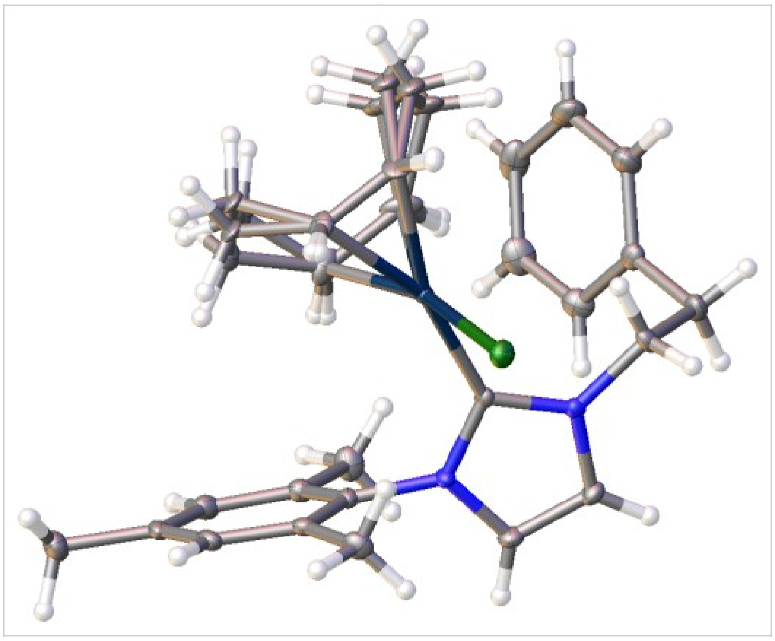

Table 10S Crystal data and structure refinement for 1e.

Identification code	sbd1613
Empirical formula	$C_{28}H_{34}ClIrN_2$
Formula weight	626.22
Temperature/K	110.05(10)
Crystal system	triclinic
Space group	P-1
a/Å	9.7423(3)
b/Å	11.0660(4)
c/Å	12.1673(4)
α/°	100.717(3)
β/°	104.685(3)
γ/°	96.543(3)
Volume/Å^3	1228.71(8)
Z	2

ρ_{calc}g/cm^3	1.693
μ/mm^{-1}	11.642
F(000)	620.0
Crystal size/mm^3	0.2421 × 0.1593 × 0.1367
Radiation	CuKα (λ = 1.54184)
2Θ range for data collection/°	7.706 to 142.354
Index ranges	-11 ≤ h ≤ 11, -10 ≤ k ≤ 13, -14 ≤ l ≤ 13
Reflections collected	7908
Independent reflections	4638 [R_{int} = 0.0146, R_{sigma} = 0.0200]
Data/restraints/parameters	4638/0/305
Goodness-of-fit on F^2	1.085
Final R indexes [I>=2σ (I)]	R_1 = 0.0167, wR_2 = 0.0413
Final R indexes [all data]	R_1 = 0.0172, wR_2 = 0.0415
Largest diff. peak/hole / e Å^{-3}	0.56/-1.03

Table S9 Fractional Atomic Coordinates (×10^4) and Equivalent Isotropic Displacement Parameters (Å^2×10^3) for sbd1613. U_{eq} is defined as 1/3 of of the trace of the orthogonalised U_{IJ} tensor.

Atom	*x*	*y*	*z*	U(eq)
Ir1	6209.9(2)	7775.6(2)	7962.6(2)	9.28(4)
Cl1	6641.4(6)	9158.8(5)	9793.4(5)	15.30(11)
N2	8886(2)	6621.7(18)	8864.5(17)	11.8(4)
N1	9501(2)	8466.2(18)	8641.9(16)	11.3(4)
C11	8325(2)	7591(2)	8465.7(19)	9.9(4)
C12	8078(3)	5421(2)	8784(2)	12.8(5)
C13	7318(3)	5263(2)	9594(2)	14.2(5)

C21	3913(3)	7391(2)	7747(2)	17.2(5)
C24	5676(3)	6171(3)	6597(2)	21.2(6)
C6	9626(3)	9015(2)	6297(2)	16.5(5)
C17	8116(3)	4432(2)	7901(2)	16.0(5)
C9	10758(3)	8067(2)	9148(2)	14.5(5)
C14	6490(3)	4096(2)	9437(2)	16.1(5)
C25	5972(3)	7243(3)	6163(2)	23.5(6)
C15	6463(3)	3097(2)	8540(2)	16.8(5)
C10	10370(3)	6914(2)	9288(2)	15.5(5)
C16	7303(3)	3274(2)	7797(2)	18.1(5)
C1	9959(3)	7819(3)	6061(2)	20.1(5)
C8	9462(3)	9694(2)	8356(2)	15.1(5)
C28	4198(3)	8453(2)	7335(2)	18.5(5)
C18	7432(3)	6294(2)	10626(2)	19.5(5)
C7	10292(3)	9884(2)	7471(2)	17.8(5)
C5	8638(3)	9380(3)	5425(2)	23.1(6)
C20	8990(3)	4603(3)	7062(2)	22.4(6)
C3	8357(3)	7396(3)	4121(2)	27.9(7)
C4	8018(3)	8581(3)	4345(2)	27.7(6)
C2	9320(3)	7009(3)	4983(2)	25.4(6)
C19	5571(3)	1836(3)	8391(3)	25.3(6)
C23	4284(6)	5335(5)	6576(5)	19.4(10)
C30	4053(9)	5632(8)	6187(7)	19.4(10)
C22	3156(12)	6149(11)	6835(8)	16.7(15)
C29	3376(18)	6054(17)	7165(12)	16.7(15)

C26	4638(7)	7592(6)	5387(5)	23.7(11)
C31	5081(10)	8115(9)	5511(8)	23.7(11)
C27	4054(16)	8591(13)	6114(16)	23(2)
C32	3750(20)	8320(20)	5980(20)	23(2)

Table S10 Anisotropic Displacement Parameters (Å^2×10^3) for sbd1613. The Anisotropic displacement factor exponent takes the form: $-2\pi^2[h^2a^{*2}U_{11}+2hka^*b^*U_{12}+…]$.

Atom	U_{11}	U_{22}	U_{33}	U_{23}	U_{13}	U_{12}
Ir1	8.40(6)	9.52(6)	10.00(6)	3.34(4)	1.76(4)	1.78(4)
Cl1	16.4(3)	13.1(3)	15.2(3)	0.1(2)	4.9(2)	1.7(2)
N2	9.4(10)	12.2(10)	14.3(9)	5.7(8)	1.9(8)	1.9(8)
N1	12(1)	11.3(9)	9.7(9)	2.8(7)	1.7(7)	0.8(8)
C11	9.3(11)	11.6(11)	9(1)	3.0(8)	3.3(8)	0.4(9)
C12	11.2(11)	11.5(11)	14.5(11)	4.8(9)	0.5(9)	1.2(9)
C13	13.4(12)	16.8(12)	13.1(11)	7.1(9)	1.5(9)	4.6(9)
C21	10.1(12)	17.7(12)	23.7(13)	3.1(10)	5.6(10)	2.6(10)
C24	17.6(13)	21.7(13)	18.5(13)	-8.4(10)	6.4(10)	-1.9(11)
C6	16.5(12)	19.2(12)	14.4(12)	5.5(10)	6.1(10)	-1.2(10)
C17	16.5(12)	16.7(12)	14.8(11)	5.5(9)	3.1(10)	2.9(10)
C9	9.4(11)	17.1(12)	15.6(11)	3.2(9)	1.7(9)	1.1(9)
C14	14.7(12)	19.2(13)	17.2(12)	11.6(10)	4.1(10)	3.5(10)
C25	23.8(14)	40.7(17)	8.9(11)	6.0(11)	4.7(10)	15.9(13)
C15	13.9(12)	13.6(12)	21.1(12)	9.3(10)	-1.9(10)	0.9(10)
C10	11.5(12)	17.1(12)	17.5(12)	5.6(9)	1.4(9)	4.6(9)
C16	20.6(13)	14.9(12)	16.0(12)	3.0(9)	0.8(10)	2.7(10)

C1	22.1(14)	24.1(14)	18.8(13)	7.1(10)	11.0(11)	7.2(11)
C8	21.1(13)	10.0(11)	14.4(11)	4.3(9)	4.8(10)	1.7(9)
C28	10.3(12)	18.7(13)	27.2(14)	7.3(10)	1.9(10)	9.8(10)
C18	25.0(14)	20.7(13)	15.6(12)	6.3(10)	8.5(10)	4.7(11)
C7	19.6(13)	16.9(12)	16.1(12)	6.3(10)	4.3(10)	-2.5(10)
C5	27.3(15)	21.8(13)	20.0(13)	9.4(11)	3.9(11)	1.7(11)
C20	26.8(15)	20.7(13)	21.3(13)	3(1)	11.3(11)	2.4(11)
C3	34.4(17)	32.2(16)	13.8(13)	-0.4(11)	11.3(12)	-8.6(13)
C4	28.3(15)	37.8(17)	15.2(13)	11.8(12)	1.4(11)	-1.2(13)
C2	33.5(16)	24.9(14)	21.0(14)	1.1(11)	16.4(12)	4.8(12)
C19	26.9(15)	18.7(13)	28.1(15)	10.5(11)	2.8(12)	-2.3(11)
C23	20(2)	14(3)	20(3)	0.4(17)	2(2)	-0.2(18)
C30	20(2)	14(3)	20(3)	0.4(17)	2(2)	-0.2(18)
C22	6(4)	18(2)	21(5)	6(3)	-4(4)	-2(2)
C29	6(4)	18(2)	21(5)	6(3)	-4(4)	-2(2)
C26	18(3)	38(4)	14.6(19)	14(3)	-1(2)	5(2)
C31	18(3)	38(4)	14.6(19)	14(3)	-1(2)	5(2)
C27	13(7)	24(6)	26(4)	10(5)	-6(4)	2(4)
C32	13(7)	24(6)	26(4)	10(5)	-6(4)	2(4)

Table S11 Bond Lengths for sbd1613.

Atom	Atom	Length/Å	Atom	Atom	Length/Å
Ir1	Cl1	2.3648(6)	C6	C1	1.394(4)
Ir1	C11	2.041(2)	C6	C7	1.504(3)
Ir1	C21	2.168(2)	C6	C5	1.395(4)

Ir1	C24	2.107(2)	C17	C16	1.394(4)
Ir1	C25	2.104(2)	C17	C20	1.509(3)
Ir1	C28	2.185(2)	C9	C10	1.343(3)
N2	C11	1.369(3)	C14	C15	1.394(4)
N2	C12	1.439(3)	C25	C26	1.536(6)
N2	C10	1.384(3)	C25	C31	1.563(9)
N1	C11	1.357(3)	C15	C16	1.389(4)
N1	C9	1.384(3)	C15	C19	1.511(3)
N1	C8	1.465(3)	C1	C2	1.391(4)
C12	C13	1.397(3)	C8	C7	1.532(3)
C12	C17	1.395(3)	C28	C27	1.49(2)
C13	C14	1.396(3)	C28	C32	1.56(3)
C13	C18	1.502(3)	C5	C4	1.384(4)
C21	C28	1.388(4)	C3	C4	1.384(4)
C21	C22	1.565(12)	C3	C2	1.386(4)
C21	C29	1.486(18)	C23	C22	1.553(14)
C24	C25	1.414(4)	C30	C29	1.53(2)
C24	C23	1.544(6)	C26	C27	1.54(2)
C24	C30	1.542(9)	C31	C32	1.57(3)

Table S12 Bond Angles for sbd1613.

Atom	Atom	Atom	Angle/˚	Atom	Atom	Atom	Angle/˚
C11	Ir1	Cl1	87.05(6)	C30	C24	Ir1	113.1(3)
C11	Ir1	C21	158.38(10)	C1	C6	C7	120.9(2)
C11	Ir1	C24	93.11(10)	C1	C6	C5	118.4(2)

C11	Ir1	C25	95.36(10)	C5	C6	C7	120.7(2)
C11	Ir1	C28	164.24(10)	C12	C17	C20	121.5(2)
C21	Ir1	Cl1	92.51(7)	C16	C17	C12	118.2(2)
C21	Ir1	C28	37.17(10)	C16	C17	C20	120.3(2)
C24	Ir1	Cl1	163.76(8)	C10	C9	N1	106.6(2)
C24	Ir1	C21	81.39(10)	C15	C14	C13	121.9(2)
C24	Ir1	C28	94.24(11)	C24	C25	Ir1	70.49(14)
C25	Ir1	Cl1	156.91(9)	C24	C25	C26	114.1(3)
C25	Ir1	C21	93.46(11)	C24	C25	C31	136.8(4)
C25	Ir1	C24	39.24(11)	C26	C25	Ir1	114.5(3)
C25	Ir1	C28	81.71(10)	C31	C25	Ir1	109.8(4)
C28	Ir1	Cl1	89.75(7)	C14	C15	C19	121.1(2)
C11	N2	C12	125.5(2)	C16	C15	C14	118.5(2)
C11	N2	C10	110.8(2)	C16	C15	C19	120.3(2)
C10	N2	C12	123.5(2)	C9	C10	N2	107.2(2)
C11	N1	C9	111.6(2)	C15	C16	C17	121.6(2)
C11	N1	C8	124.9(2)	C2	C1	C6	120.8(3)
C9	N1	C8	123.6(2)	N1	C8	C7	112.3(2)
N2	C11	Ir1	127.67(17)	C21	C28	Ir1	70.76(14)
N1	C11	Ir1	127.86(17)	C21	C28	C27	129.7(5)
N1	C11	N2	103.86(19)	C21	C28	C32	117.3(7)
C13	C12	N2	120.1(2)	C27	C28	Ir1	108.5(7)
C17	C12	N2	117.9(2)	C32	C28	Ir1	112.7(11)
C17	C12	C13	122.0(2)	C6	C7	C8	112.8(2)
C12	C13	C18	121.4(2)	C4	C5	C6	120.9(3)

C14	C13	C12	117.7(2)	C4	C3	C2	119.8(3)
C14	C13	C18	120.9(2)	C3	C4	C5	120.2(3)
C28	C21	Ir1	72.07(14)	C3	C2	C1	119.9(3)
C28	C21	C22	118.1(4)	C24	C23	C22	110.3(6)
C28	C21	C29	133.2(5)	C29	C30	C24	109.4(8)
C22	C21	Ir1	112.6(5)	C23	C22	C21	110.6(7)
C29	C21	Ir1	108.4(7)	C21	C29	C30	111.8(12)
C25	C24	Ir1	70.27(15)	C25	C26	C27	109.9(7)
C25	C24	C23	134.3(3)	C25	C31	C32	110.2(11)
C25	C24	C30	111.3(4)	C28	C27	C26	114.2(11)
C23	C24	Ir1	111.5(2)	C28	C32	C31	109.3(14)

Table S13 Torsion Angles for sbd1613.

A	B	C	D	Angle/˚	A	B	C	D	Angle/˚
Ir1	C21	C28	C27	98.4(9)	C6	C5	C4	C3	1.0(4)
Ir1	C21	C28	C32	106.2(12)	C17	C12	C13	C14	-5.2(4)
Ir1	C21	C22	C23	15.6(7)	C17	C12	C13	C18	172.4(2)
Ir1	C21	C29	C30	-41.3(8)	C9	N1	C11	Ir1	170.82(16)
Ir1	C24	C25	C26	-108.9(3)	C9	N1	C11	N2	-0.7(3)
Ir1	C24	C25	C31	-98.5(6)	C9	N1	C8	C7	61.3(3)
Ir1	C24	C23	C22	40.4(6)	C14	C15	C16	C17	-2.6(4)
Ir1	C24	C30	C29	-23.0(8)	C25	C24	C23	C22	-42.6(7)
Ir1	C25	C26	C27	17.3(8)	C25	C24	C30	C29	-99.8(8)
Ir1	C25	C31	C32	-44.2(10)	C25	C26	C27	C28	-35.4(9)
Ir1	C28	C27	C26	35.6(8)	C25	C31	C32	C28	39.3(14)

Ir1	C28	C32	C31	-17.1(14)	C10	N2	C11	Ir1	-170.73(17)
N2	C12	C13	C14	176.6(2)	C10	N2	C11	N1	0.8(3)
N2	C12	C13	C18	-5.9(3)	C10	N2	C12	C13	103.9(3)
N2	C12	C17	C16	-178.2(2)	C10	N2	C12	C17	-74.5(3)
N2	C12	C17	C20	0.8(4)	C1	C6	C7	C8	-85.8(3)
N1	C9	C10	N2	0.2(3)	C1	C6	C5	C4	-0.9(4)
N1	C8	C7	C6	65.3(3)	C8	N1	C11	Ir1	-8.2(3)
C11	N2	C12	C13	-81.0(3)	C8	N1	C11	N2	-179.7(2)
C11	N2	C12	C17	100.7(3)	C8	N1	C9	C10	179.4(2)
C11	N2	C10	C9	-0.7(3)	C28	C21	C22	C23	96.7(5)
C11	N1	C9	C10	0.3(3)	C28	C21	C29	C30	41.1(13)
C11	N1	C8	C7	-119.8(2)	C18	C13	C14	C15	-174.6(2)
C12	N2	C11	Ir1	13.6(3)	C7	C6	C1	C2	178.7(2)
C12	N2	C11	N1	-174.8(2)	C7	C6	C5	C4	-179.8(2)
C12	N2	C10	C9	175.1(2)	C5	C6	C1	C2	-0.2(4)
C12	C13	C14	C15	2.9(4)	C5	C6	C7	C8	93.1(3)
C12	C17	C16	C15	0.5(4)	C20	C17	C16	C15	-178.5(2)
C13	C12	C17	C16	3.5(4)	C4	C3	C2	C1	-1.0(4)
C13	C12	C17	C20	-177.5(2)	C2	C3	C4	C5	0.0(4)
C13	C14	C15	C16	0.9(4)	C19	C15	C16	C17	178.8(2)
C13	C14	C15	C19	179.4(2)	C23	C24	C25	Ir1	101.2(4)
C21	C28	C27	C26	-44.3(12)	C23	C24	C25	C26	-7.7(5)
C21	C28	C32	C31	-96.3(10)	C30	C24	C25	Ir1	107.9(4)
C24	C25	C26	C27	95.9(7)	C30	C24	C25	C31	9.4(7)
C24	C25	C31	C32	38.0(12)	C22	C21	C28	Ir1	-106.6(5)

C24	C23	C22	C21	-35.7(8)	C22	C21	C28	C27	-8.2(11)
C24	C30	C29	C21	42.6(10)	C29	C21	C28	Ir1	-98.8(10)
C6	C1	C2	C3	1.1(4)	C29	C21	C28	C32	7.5(15)

Table S14 Hydrogen Atom Coordinates (Å×10[4]) and Isotropic Displacement Parameters (Å[2]×10[3]) for sbd1613.

Atom	*x*	*y*	*z*	U(eq)
H21	3623	7525	8469	21
H21A	3694	7597	8496	21
H24	6475	5700	6656	25
H24A	6354	5581	6614	25
H9	11689	8511	9351	17
H14	5941	3982	9945	19
H25	6855	7335	5919	28
H25A	6920	7290	6020	28
H10	10984	6408	9610	19
H16	7323	2604	7216	22
H1	10617	7560	6631	24
H8A	9874	10337	9064	18
H8B	8469	9782	8042	18
H28	4118	9218	7851	22
H28A	4086	9239	7801	22
H18A	8427	6575	11054	29
H18B	6916	5989	11121	29
H18C	7028	6975	10361	29
H7A	10328	10738	7380	21
H7B	11273	9754	7770	21

H5	8392	10170	5570	28
H20A	8736	5286	6714	34
H20B	8793	3854	6464	34
H20C	9996	4777	7475	34
H3	7939	6860	3395	33
H4	7373	8842	3768	33
H2	9538	6208	4839	30
H19A	6053	1414	8959	38
H19B	5448	1348	7622	38
H19C	4646	1946	8497	38
H23A	4503	4837	7155	23
H23B	3890	4772	5816	23
H30A	3593	5921	5502	23
H30B	3915	4728	5982	23
H22A	2482	5687	7135	20
H22B	2619	6340	6119	20
H29A	2338	5926	6846	20
H29B	3594	5547	7737	20
H26A	3901	6857	5044	28
H26B	4890	7909	4760	28
H31A	4754	7744	4682	28
H31B	5684	8914	5626	28
H27A	4561	9407	6139	27
H27B	3044	8557	5727	27
H32A	3400	9063	5798	27

H32B	2973	7612	5619	27

Table S15 Atomic Occupancy for sbd1613.

Atom	*Occupancy*	Atom	*Occupancy*	Atom	*Occupancy*
H21	0.576(5)	H21A	0.424(5)	H24	0.576(5)
H24A	0.424(5)	H25	0.576(5)	H25A	0.424(5)
H28	0.576(5)	H28A	0.424(5)	C23	0.576(5)
H23A	0.576(5)	H23B	0.576(5)	C30	0.424(5)
H30A	0.424(5)	H30B	0.424(5)	C22	0.576(5)
H22A	0.576(5)	H22B	0.576(5)	C29	0.424(5)
H29A	0.424(5)	H29B	0.424(5)	C26	0.576(5)
H26A	0.576(5)	H26B	0.576(5)	C31	0.424(5)
H31A	0.424(5)	H31B	0.424(5)	C27	0.576(5)
H27A	0.576(5)	H27B	0.576(5)	C32	0.424(5)
H32A	0.424(5)	H32B	0.424(5)		

Experimental

Single crystals of $C_{28}H_{34}ClIrN_2$ [sbd1613] were selected and placed in a micromount on a SuperNova, Dual, Cu at zero, Eos diffractometer. The crystal was kept at 110.05(10) K during data collection. Using Olex2 [1], the structure was solved with the ShelXT [2] structure solution program using Direct Methods and refined with the ShelXL [3] refinement package using Least Squares minimisation.

1. Dolomanov, O.V., Bourhis, L.J., Gildea, R.J, Howard, J.A.K. & Puschmann, H. (2009), J. Appl. Cryst. 42, 339-341.
2. Sheldrick, G.M. (2015). Acta Cryst. A71, 3-8.
3. Sheldrick, G.M. (2015). Acta Cryst. C71, 3-8.

Crystal structure determination of [sbd1613]

Crystal Data for $C_{28}H_{34}ClIrN_2$ (M =626.22 g/mol): triclinic, space group P-1 (no. 2), a = 9.7423(3) Å, b = 11.0660(4) Å, c = 12.1673(4) Å, α = 100.717(3)°, β = 104.685(3)°, γ = 96.543(3)°, V = 1228.71(8) Å^3, Z = 2, T = 110.05(10) K, μ(CuKα) = 11.642 mm^{-1}, $Dcalc$ = 1.693 g/cm^3, 7908 reflections measured (7.706° ≤ 2Θ ≤ 142.354°), 4638 unique (R_{int} = 0.0146, R_{sigma} = 0.0200) which were used in all calculations. The final R_1 was 0.0167 (I > 2σ(I)) and wR_2 was 0.0415 (all data).

Refinement model description Number of restraints - 0, number of constraints - unknown.

Details:

```
1. Fixed Uiso
 At 1.2 times of:
  All C(H) groups, All C(H,H) groups
 At 1.5 times of:
  All C(H,H,H) groups
2. Uiso/Uaniso restraints and constraints
```

```
Uanis(C23) = Uanis(C30)
Uanis(C26) = Uanis(C31)
Uanis(C27) = Uanis(C32)
Uanis(C22) = Uanis(C29)
3. Others
 Sof(H21A)=Sof(H24A)=Sof(H25A)=Sof(H28A)=Sof(C30)=Sof(H30A)=Sof(H30B)=Sof(C29)=
 Sof(H29A)=Sof(H29B)=Sof(C31)=Sof(H31A)=Sof(H31B)=Sof(C32)=Sof(H32A)=Sof(H32B)=
 1-FVAR(1)
 Sof(H21)=Sof(H24)=Sof(H25)=Sof(H28)=Sof(C23)=Sof(H23A)=Sof(H23B)=Sof(C22)=
 Sof(H22A)=Sof(H22B)=Sof(C26)=Sof(H26A)=Sof(H26B)=Sof(C27)=Sof(H27A)=Sof(H27B)=
 FVAR(1)
4.a Ternary CH refined with riding coordinates:
 C21(H21), C21(H21A), C24(H24), C24(H24A), C25(H25), C25(H25A), C28(H28),
 C28(H28A)
4.b Secondary CH2 refined with riding coordinates:
 C8(H8A,H8B), C7(H7A,H7B), C23(H23A,H23B), C30(H30A,H30B), C22(H22A,H22B),
 C29(H29A,H29B), C26(H26A,H26B), C31(H31A,H31B), C27(H27A,H27B), C32(H32A,H32B)
4.c Aromatic/amide H refined with riding coordinates:
 C9(H9), C14(H14), C10(H10), C16(H16), C1(H1), C5(H5), C3(H3), C4(H4), C2(H2)
4.d Idealised Me refined as rotating group:
 C18(H18A,H18B,H18C), C20(H20A,H20B,H20C), C19(H19A,H19B,H19C)
```

This report has been created with Olex2, compiled on 2016.02.19 svn.r3266 for OlexSys.

References

1. J. Choudhury, S. Podder and S. Roy, *Journal of the American Chemical Society*, 2005, **127**, 6162-6163.
2. L. Zhu, P. Guo, G. Li, J. Lan, R. Xie and J. You, *Journal of Organic Chemistry*, 2007, **72**, 8535-8538.
3. A. J. Ruddlesden, R. E. Mewis, G. G. R. Green, A. C. Whitwood and S. B. Duckett, *Organometallics*, 2015, **34**, 2997-3006.
4. C. Nieto-Oberhuber, M. P. Munoz, S. Lopez, E. Jimenez-Nunez, C. Nevado, E. Herrero-Gomez, M. Raducan and A. M. Echavarren, *Chemistry - A European Journal*, 2006, **12**, 1677-1693.
5. B. Eguillor, M. A. Esteruelas, M. Oliván and M. Puerta, *Organometallics*, 2008, **27**, 445-450.
6. L. S. Lloyd, R. W. Adams, M. Bernstein, S. Coombes, S. B. Duckett, G. G. R. Green, R. J. Lewis, R. E. Mewis and C. J. Sleigh, *Journal of the American Chemical Society*, 2012, **134**, 12904-12907.
7. R. V. Shchepin, L. Jaigirdar, T. Theis, W. S. Warren, B. M. Goodson and E. Y. Chekmenev, *J. Phys. Chem. C*, 2017, **121**, 28425-28434.
